# The *Streptococcus* virulence protein PepO triggers anti-tumor immune responses by reprograming tumor-associated macrophages in a mouse triple negative breast cancer model

**DOI:** 10.1186/s13578-023-01153-w

**Published:** 2023-11-04

**Authors:** Bichen Liu, Jun Huang, Jiangming Xiao, Wenlong Xu, Hong Zhang, Yuan Yuan, Yibing Yin, Xuemei Zhang

**Affiliations:** 1 Department of Laboratory Medicine, Key Laboratory of Diagnostic Medicine (Ministry of Education), Chongqing Medical University, Chongqing, 400016 China; 2 Department of Laboratory Medicine, The First Affiliated Hospital of Chongqing Medical University, Chongqing, 400016 China; 3 Department of Laboratory Medicine, The Affiliated Hospital of North Sichuan Medical College, Nanchong, China; 4 Department of Laboratory Medicine and Translational Medicine Research Center, North Sichuan Medical College, Nanchong, 637000 China

**Keywords:** Streptococcus pneumoniae endopeptidase O(PepO), Triple negative breast cancer (TNBC), Tumor associated macrophages reprograming, Tumor microenvironment, Cancer immunotherapy, Immunoregulatory molecule

## Abstract

**Background:**

The efficacy of current surgery and chemotherapy for triple negative breast cancer (TNBC) is limited due to heterogenous and immunosuppressive tumor microenvironment (TME). Tumor associated macrophages (TAMs), which are regarded as an M2 tumor-promoting phenotype, are crucial in the development of the immunosuppressive TME. Targeting TAM reprograming is a promising strategy in anti-tumor therapy since reprogramming techniques provide the opportunity to actively enhance the antitumor immunological activity of TAM in addition to eliminating their tumor-supportive roles, which is rarely applied in TNBC clinically. However, how to drive M2 macrophages reprogramming into M1 with high potency remains a challenge and the molecular mechanisms how M2 macrophages polarized into M1 are poorly understood. Here, we identified a new immunoregulatory molecular PepO that was served as an immunoregulatory molecule governed the transformation of tumor-promoting M2 to tumor-inhibitory M1 cells and represented an effective anti-tumor property.

**Results:**

At the present study, we identified a new immunoregulatory molecular PepO, as a harmless immunoregulatory molecule, governed the transformation of tumor-promoting M2 to tumor-inhibitory M1 cells efficiently. PepO-primed M2 macrophages decreased the expression of tumor-supportive molecules like Arg-1, Tgfb, Vegfa and IL-10, and increased the expression of iNOS, Cxcl9, Cxcl10, TNF-α and IL-6 to inhibit TNBC growth. Moreover, PepO enhanced the functions of macrophages related to cell killing, phagocytosis and nitric oxide biosynthetic process, thereby inhibiting the development of tumors in vivo and in vitro. Mechanistically, PepO reprogramed TAMs toward M1 by activating PI3K-AKT-mTOR pathway via TLR4 and suppressed the function of M2 by inhibiting JAK2-STAT3 pathway via TLR2. The PI3K inhibitor LY294002 abrogated the role of PepO in switching M2 macrophages into M1 and in inhibiting TNBC growth in vivo. And PepO failed to govern the M2 macrophages to reprogram into M1 macrophages and inhibit TNBC when TLR2 or TLR4 was deficient. Moreover, PepO enhanced the antitumor activity of doxorubicin and the combination exerted a synergistic effect on TNBC suppression.

**Conclusions:**

Our research identified a possible macrophage-based TNBC immunotherapeutic approach and suggested a novel anticancer immunoregulatory molecular called PepO.

**Supplementary Information:**

The online version contains supplementary material available at 10.1186/s13578-023-01153-w.

## Background

Triple negative breast cancer (TNBC) is defined as the absence of tumor tissue expression of the estrogen receptor (ER), progesterone receptor (PR) and the human epidermal growth factor receptor 2 (HER2). TNBC is the most aggressive, metastatic and remarkably drug-resistant subtype of breast cancer particularly due to the lack of specific and targeted therapeutics [1]. Global cases of TNBC account for 20% of all breast cancers [2] and 83% of disproportionate deaths compared to all other subtypes [3]. Chemotherapy is the only treatment option for TNBC but effectiveness is poor [4, 5] due to the heterogeneity of oncogenic drivers [6–8] and drug resistance [9–11]. The current arsenal of TNBC chemotherapeutics includes cisplatin, anthracycline, paclitaxel, tamoxifen and doxorubicin [12]. Therefore, new therapeutic strategies are urgently needed.

The distinct tumor microenvironment (TME) of TNBC is highly complex and heterogeneous and is characterized by immunosuppression, angiogenesis, inhibition of apoptosis, stimulation of proliferation and drug resistance [13, 14]. TME is composed of tumor cells, infiltrating immune cells such as macrophages, dendritic cells (DC) and lymphocytes, cancer-associated stromal cells including fibroblasts, endothelial cells and lipocytes that associate with the extracellular matrix and signaling molecules [15, 16]. Cancer cells are more likely to metastasize as a result of alterations in TME biological components brought on by reciprocal communication between stromal cells, immune cells and cancer cells [17]. Tumor-associated macrophages (TAM) are one of the most significant stromal components of the TME, and they are biologically heterogeneous and promote TNBC progression by releasing inhibitory cytokines that repress tumor infiltrating lymphocyte functions, promote Treg conversions and are closely linked to the progression of malignant tumors. The resident macrophage M0 phenotype in the TME may be polarized into either pro-inflammatory M1 or anti-inflammatory M2 macrophages. M1 cells exhibit tumoricidal action via iNOS (inducible nitric oxide synthase) that metabolizes arginine to produce nitric oxide that diffuses into adjacent tumor cells resulting in cell death [18, 19]. M1 macrophages also secret several proinflammatory cytokines including TNF-α, IFN-γ, IL-6 and IL-1β [20] to suppress even kill cancer cells. In contrast, M2 macrophages promote tumor development via proangiogenic factors and immunosuppressive cytokines like TGF-β and IL-10 [21]. While macrophage polarization is frequently discussed as occurring at a specific time, the M1 and M2 phenotypes are interconvertible depending on environmental factors such as cytokine and growth factor release, inflammation, infection, injury and hypoxia [22]. Phenotypic reprograming is a current focus of tumor immunotherapy and can be beneficial for diagnostic, prognostic and therapeutic targets.

Toll-like receptors (TLR) are pattern-recognition receptors and are crucial in triggering both innate and adaptive immunity [23]. TLR agonists have been used to directly reprogram macrophages and this approach has been successful in a murine model using the R848 (Resiquimod) nanoparticles with imidazoquinoline TLR7/8 agonist [24]. Another TLR7/8 dual agonist ISACs conjugated to tumor-targeting antibodies induced a robust localized activation of macrophages and DCs leading to tumor clearance and immunological memory [25]. Moreover, the TLR7 ligand imiquimod coupled with the TLR8 agonist motolimod displayed anti-tumor activity and triggered a strong innate and adaptive immunity. However, patients rarely benefit therapeutically from imiquimod and motolimod. The specific targeting of the TLR with immunoregulatory molecules are a promising approach and more effort and attempts should be taken.

We previously reported that the *Streptococcus pneumoniae* endopeptidase O (PepO) virulence protein induces innate and adaptive immune responses that acts via TLR2/TLR4 [26]. In the present study we verified that PepO acts as a TLR2/4 dual ligand agonist resulting in switching M2 macrophage to the tumoricidal M1 macrophage by activating PI3K-AKT-mTOR and inhibiting JAK2-STAT3 pathway and enhanced the anti-tumor property of the chemotherapeutic drug doxorubicin. Our findings reveal a pivotal role for PepO as an immune modulator in promoting M1 polarization and the development of a novel macrophage-based therapeutic for TNBC immunotherapy.

## Methods

### Mice

Female C57BL/6 J mice, 6–8 week, were obtained from Beijing Huafukang Bioscience (Beijing, China) and bred at Chongqing Medical University. TLR2, TLR4 and TLR2/4 deficient mice were purchased from the Jackson Laboratory (Bar Harbor, ME). Mice were anesthetized by 1.5% pentobarbital sodium solution.

### Cell culture

Murine TNBC cell lines PY8119 and 4T1 were obtained from the American Type Culture Collection (Manassas, VA, USA) and cultured in high glucose Dulbecco’s Modified Eagle medium (DMEM) with glutamine and supplemented with 10% FBS and 100 µg/ml penicillin and streptomycin. The standard conditions for culture were 37 °C in a 5% CO_2_ humidified atmosphere (Additional file 2: Table S1).

### Tumor growth and treatments

We established the TNBC mouse model by injection of 1 × 10^6^ PY8119 cells (in 50 µL PBS) into the 4th mammary fat pad of C57BL/6 mice and TLR2^−/−^ or/and TLR4^−/−^ C57BL/6 mice. The mice were randomly allocated to experimental groups 1 week later and were then intraperitoneally (i.p) injected with the different test components every 4 days over 12 days. The experimental groups received (1) PBS (negative control) (2)1 mg PepO (3) PepO treated with protease K at 56 °C for 60 min prior to injection and (4) 1 mg BSA and LPS (equivalent control). Combination therapy experiments were performed in PY8119-bearing C57BL/6 mice and treated with PBS, PepO, doxorubicin (5 mg/kg) and PepO/doxorubicin i.p. Clodronate liposomes were used to deplete macrophages in vivo, 100 μl clodronate liposomes or control liposomes (10 mg/ml) were injected through tail vein of PY8119-bearing mice 24 h prior to PepO or PBS treatments as described above and was repeated every 5 d thereafter to maintain macrophage depletion. The PI3K inhibitor LY294002 (50 mg/kg) or the STAT3 activator colivelin (1 mg/kg) was i.p. administrated 24 h before the treatment of PepO or PBS, and injected every 2 days. The size of tumors was measured using a caliper and the resulting tumors were excised and weighed. The tumor volume was calculated using the equation L × W^2^ × 0.5 where L = length and W = width measured by two observers blinded to the group allocations.

### Isolation and polarization of mouse BMDM

Bone marrow derived macrophages (BMDM) were isolated and differentiated using standard protocols [27, 28]. In brief, primary macrophages from bone marrow cells and were cultured 7d in DMEM containing recombinant macrophage colony-stimulating factor (20 ng/mL) to obtain M0 populations. These cells were seeded into 6-well plates at 2.5 × 10^6^ cells/well and incubated overnight. The cells were polarized to the M2 phenotype by exposure to 20 ng/mL each of IL-4 and IL-13 for 24 h and were stimulated with 5 μg/ml PepO or PBS for an additional 24 h. Differentiated BMDM and PY8119 were co-cultured in transwell: 2.5 × 10^6^ BMDMs were seeded into 6-well plates for 24 h and the 0.4 μm pore Transwell inserts (Corning) containing 5 × 10^5^ PY8119 cells were placed into the wells for 24 h. The supernatants and macrophages were collected at the indicated times for cytokine and mRNA measurements (see below).

### Preparation of conditioned medium and co-cultured with PY8119 cells

Macrophage-conditioned medium (CM) was obtained from 3 × 10^6^ differentiated BMDM cultured in DMEM complete medium for 24 h (M0 BMDM) or treated with PepO for 24 h (M0 + PepO). M2 BMDM were polarized from M0 BMDM as per above and these M2 BMDM were treated with PepO for an additional 24 h (M2 + PepO). The medium was replaced with fresh FBS-free DMEM and incubated for 24 h. The culture supernatants were collected and centrifuged at 1000 rpm for 5 min and filtered through 0.2 μm membranes and designated as M0 CM, M0 + PepO CM, M2 CM and M2 + PepO CM, respectively. Co-culture assays utilized 5 × 10^5^ PY8119 cells seeded into 6-well plates containing DMEM complete medium for 24 h. The supernatants were discarded and added DMEM with PepO, BSA + LPS and PBS or CMs and incubated 48 h under standard conditions. The PY8119 cells were collected and analyzed for apoptotic and proliferation markers (see below).

### RNA extraction and real-time quantitative PCR

Total RNA was isolated using Trizol reagent (Invitrogen) and standard cDNA synthesis reactions were carried out using a commercial reverse transcription system (Promega). The resulting cDNA was quantified using a SYBR Green real-time PCR kit (Takara). The mRNA expression levels of specific gene targets (Additional file 2: Table S2) were normalized to the levels of *Gapdh*.

### Protein extraction and western blot

Protein extraction and Western blotting were performed using standard protocols [29]. In brief, cells were lysed using RIPA buffer containing protease and phosphatase inhibitors. The proteins were separated using SDS-PAGE and electro transferred to PVDF membranes (Millipore) and incubated with primary antibodies (Additional file 2: Table S3) at 4 °C overnight followed by HRP-conjugated secondary antibodies. Proteins were visualized using an ECL Western blot analysis reagent and a ChemiDocXRS^+^ system (Biorad).

### Immunofluorescent staining

Tumor tissues were stained using fluorescently-labeled antibodies to F4/80, Arg-1, iNOS, E-cadherin, vimentin, snail and Ki-67. Briefly, 5 μm thick tumor biopsy sections were deparaffinized in xylene and rehydrated in an ethanol series. Sections were placed in antigen-retrieval solution (10 mM sodium citrate, pH 6.0) and boiled 3 min and cooled to room temperature. Tissue sections were then incubated with CY3-α-F4/80, CY5-α-Arg-1, FITC-α-iNOS, FITC-α-E-cadherin, CY3-α-vimentin, CY5-α-Snail and CY3-α-Ki-67 antibodies overnight at 4 °C. Immediately prior to analysis the slides were stained with 4'-6-diamidino-2-phenylindole (DAPI) at room temperature for 5 min. The images were captured using a Zeiss LSM800optical system.

### Isolation of single cells

Single cells were isolated from dissected tumor tissues that had been minced into small pieces and digested for 1 h at 37 °C in DMEM medium with 300 U/mL type IV collagenase and 100 U/ml DNase I. The digestion mixture was then passed through a 70 μm sieve and the filtrate cells were suspended by vigorous pipetting and washed 2 × in BMDM medium.

### Analysis of RNA-seq data

Raw data of FASTQ format were firstly processed through in-house perl scripts. Clean data were obtained by removing reads containing adapter, reads containing poly-N and low quality reads from raw data. At the same time, Q20, Q30 and GC content the clean data were calculated. All the downstream analyses were based on the clean data with high quality. Differential expression analysis of PepO treatment or control group was performed using the DESeq2 R package (1.16.1). The resulting P-values were adjusted using the Benjamini and Hochberg’s approach for controlling the false discovery rate. Genes with an adjusted p-value < 0.05 found by DESeq2 were assigned as differentially expressed. Gene Ontology (GO) enrichment analysis of differentially expressed genes was implemented by the clusterProfiler R package, in which gene length bias was corrected. GO terms with corrected P-value less than 0.05 were considered significantly enriched by differential expressed genes. We used clusterProfiler R package to test the statistical enrichment of differential expression genes in KEGG path.

### Flow cytometry

The Fc receptors of single cancer cells were blocked using anti-mouse CD16/32 and then stained with anti-mouse surface marker antibodies (Additional file 2: Table S3). Flow cytometry was performed on FACSAria Fusion platform (BD Biosciences) and data were analyzed using FlowJo software. Intracellular antigens were stained following surface antigen staining as described: cells were fixed using a commercial Cyto-Fast^™^ Fix/Perm Buffer Set kit in Fixation Buffer in the dark for 20 min. The collected cells were resuspended in Intracellular Staining Perm Wash Buffer and blocked with 2 μL CD16/32 for 30 min followed by the addition of fluorophore-conjugated α-iNOS for 30 min. The cells were washed and fixed and intracellularly labeled in 0.5 mL cell staining buffer for flow cytometry analysis (Additional file 2: Table S4).

### Wound-healing assay

PY8119 cells were inoculated in 6-well culture plates until the confluence reached 90%. After serum starvation for 24 h, a sterile pipette tip was used to scratch the monolayer. Then conditional medium including M0, M0 + PepO, M2, M2 + PepO CMs were added to each plate. The distance that cells had migrated was photographed at 0, 12, 24, 36 h.

### Statistical analysis

In pairwise comparisons, a two-tailed Student t test was used. To analyze more than two groups, a one-way ANOVA with Tukey’s multiple comparisons test was used. When comparing multiple variables that belong to two different groups, Two-way ANOVA with Tukey’s multiple comparisons test was used. Two-way repeated-measures ANOVA with Sidak’s multiple comparisons test was used in tumor growth profiles. Data was presented as mean ± SEM of the independent experiments. All statistical analysis were conducted with Prism V.8 software (GraphPad, San Diego, CA, USA). A P value < 0.05 was considered statistically significant.

## Results

### PepO reprograms TAM to the tumoricidal phenotype in vitro

**Fig. 1:**
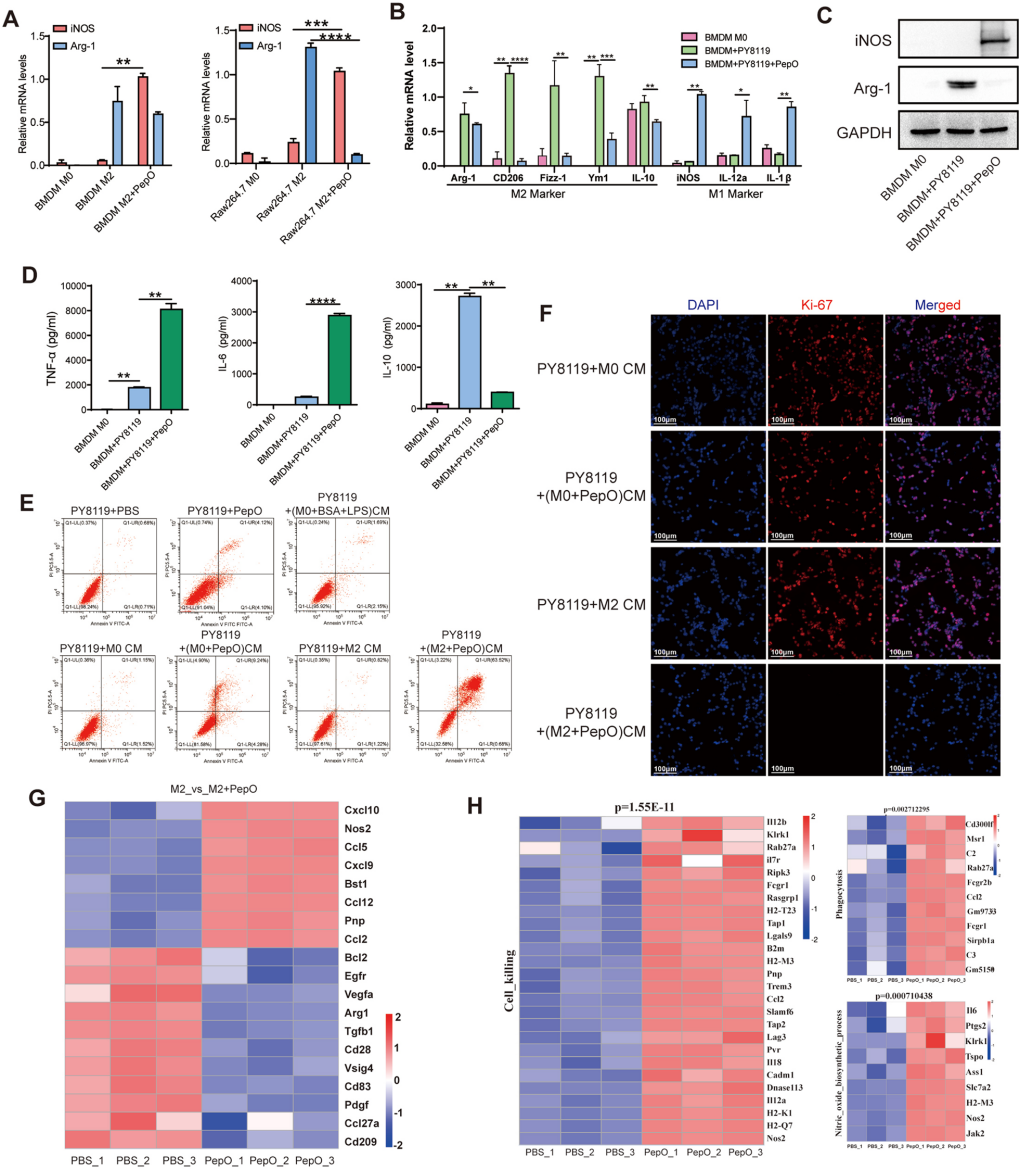
PepO re-programs TAM to the tumoricidal phenotype in vitro. **A** BMDM or Raw264.7 was polarized into M2 macrophages by IL4(20 ng/ml) and IL-13(20 ng/ml) for 24 h, and then treated with PepO for another 24 h. The transcription level of M1-associated and M2-associated markers were detected by RT-qPCR (n = 3). **B** BMDM was co-cultured with PY8119 in the presence of PepO or not. The transcription level of macrophage markers was determined by RT-qPCR. Arg-1, CD206, Fizz-1, Ym1, and IL-10 are markers of TAMs, and iNOS, IL-12a, IL-1β are markers of tumoricidal macrophages (n = 3). **C** The protein level of M1 marker iNOS and M2 marker Arg-1 in TAMs(BMDM + PY8119) and TAMs treated with PepO were evaluated by a western blot analysis. **D** The expression of pro-inflammatory cytokine TNF-α and IL-6, and anti-inflammatory cytokine IL-10 in TAMs or TAMs treated by PepO were detected using ELISA kit (n = 3). **E** PY8119 cells were co-cultured with PepO or PBS treated M0 or M2 BMDM, and the percentage of apoptotic PY8119 cells labeled with PI and Annexin V were detected by FACS. **F** Cell proliferation (Ki-67) was evaluated using Immunofluorescence detection (scale bar = 100 μm). **G** Heatmap of the DEGs that were uniquely changed in PepO treated M2 macrophages group. **H** RNA-seq heatmaps showing different gene transcripts (P < 0.05) related to cell killing, phagocytosis and Nitric oxide biosynthetic process in PepO or PBS treated M2 macrophages. Two-way ANOVA with Tukey’s multiple comparisons test was used in (A). One-way ANOVA with Tukey’s multiple comparisons test was used in (B), and (D). Bar graphs represent mean ± SEM, *P < 0.05, **P < 0.01, ***P < 0.001, ****P < 0.0001

Tumor cells assist in the reprogramming of macrophages to an M2 phenotype (TAM) that enables tumor development. The natural opposition to this process is the M1 macrophages that secrete pro-inflammatory cytokines and reactive oxygen/nitrogen species that contribute to tumor cell cytotoxicity [30]. We previously found that PepO could induce a robust innate immune response in mice in a TLR2/TLR4-dependent manner [26, 31]. We therefore examined whether PepO was functioning as an immunomodulator that allowed reprograming of the tumor-permissive M2 phenotype to the tumor-inhibitory M1 phenotype subsequently suppressed tumor growth. We added PepO to cultured M2 BMDM cells to attempt to switch their phenotype to M1. We found that PepO could reprogram M2 macrophages into M1 phenotype by M1-specific iNOS mRNA levels were increased while the M2-specific Arg1 levels were decreased (Fig. 1A). BMDM or the macrophage cell line Raw264.7 was co-cultured with PY8119 cells to mimic the TNBC microenvironment in vitro, and macrophages were polarized toward pro-tumor M2 phenotype and M2 markers Arg-1, CD206, Fizz-1, Ym1 and IL-10 were upregulated significantly (Fig. 1B and Additional file 1: Fig S1A). The addition of PepO to the co-cultures counteracted the PY8119 effects and converted the M2 macrophages into M1 macrophages as indicated by the upregulation of the M1 markers iNOS, IL12a, IL1β and the downregulation of Arg-1, CD206, Fizz-1, Ym1, and IL-10 (Fig. 1B, C and Additional file 1: Fig S1A). In addition, PepO treatment also led to significant increases in the pro-inflammatory cytokines TNF-α and IL-6 while decreasing anti-inflammatory IL-10 levels in both BMDM and Raw264.7 cells (Fig. 1D and Additional file 1: Fig S1B). Collectively, these data suggested an overwhelming effect of PepO in governing repolarization from M2 to M1.

We next evaluated whether PepO-primed M2 macrophages gained anti-cancer capabilities. PY8119 cells co-cultured with PepO-primed BMDM CM contained significantly and greater levels of apoptotic cells (about 60%) compared to co-cultures with M0 or M2 BMDM CM (about 1%) (Fig. 1E and Additional file 1: Fig S1C). Similar results were observed using 4T1 tumor cell co-cultures with CMs (Additional file 1: Fig S1D). Moreover, the level of cleaved caspase-3, a specific marker of cell apoptosis, was enhanced in PepO-primed BMDM CM co-cultures (Additional file 1: Fig S1E). In addition, PepO-primed M0 or M2 CM macrophages robustly suppressed PY8119 cell proliferation in vitro (Fig. 1F). Interestingly, the anti-cancer property in PepO-primed M2 macrophages was greater than PepO-primed M0 macrophages (compare Figs. 1E, Additional file 1: Fig S1C, D).

These data promoted us to hypothesize that PepO-primed M2 BMDM exert antitumor immunity via enhanced phagocytosis and/or tumoricidal functions. Subsequently, we systematically analyzed the reprogramming effect of PepO by assessing total transcriptome changes of M2 BMDM treated with PBS or PepO. The results showed that PepO treatment caused significant upregulation of M1-associated functional markers including Nos2, Cxcl9, Cxcl10 and Ccl5, and downregulation of the M2-associated functional markers Arg-1, Vegfa, Tgfb1 and CD209 (Fig. 1G). Rt-qPCR was used to quantify and verify the mRNA of RNA-seq and the tendency agreed with the RAN-seq. (Additional file 1: Fig S1F). These data systematically demonstrated that PepO reversed M2 macrophage into M1 phenotype. Furthermore, PepO-primed M2 BMDM showed enhanced transcription of gene clusters related to cell killing, phagocytosis and nitric oxide biosynthetic process that are closely related to antitumor functions of macrophages (Fig. 1H). These data provided further evidence that PepO altered the M2 phenotype to the anti-tumor M1 macrophage phenotype.

### *PepO treatment suppresses TNBC growth *in vivo

**Fig. 2:**
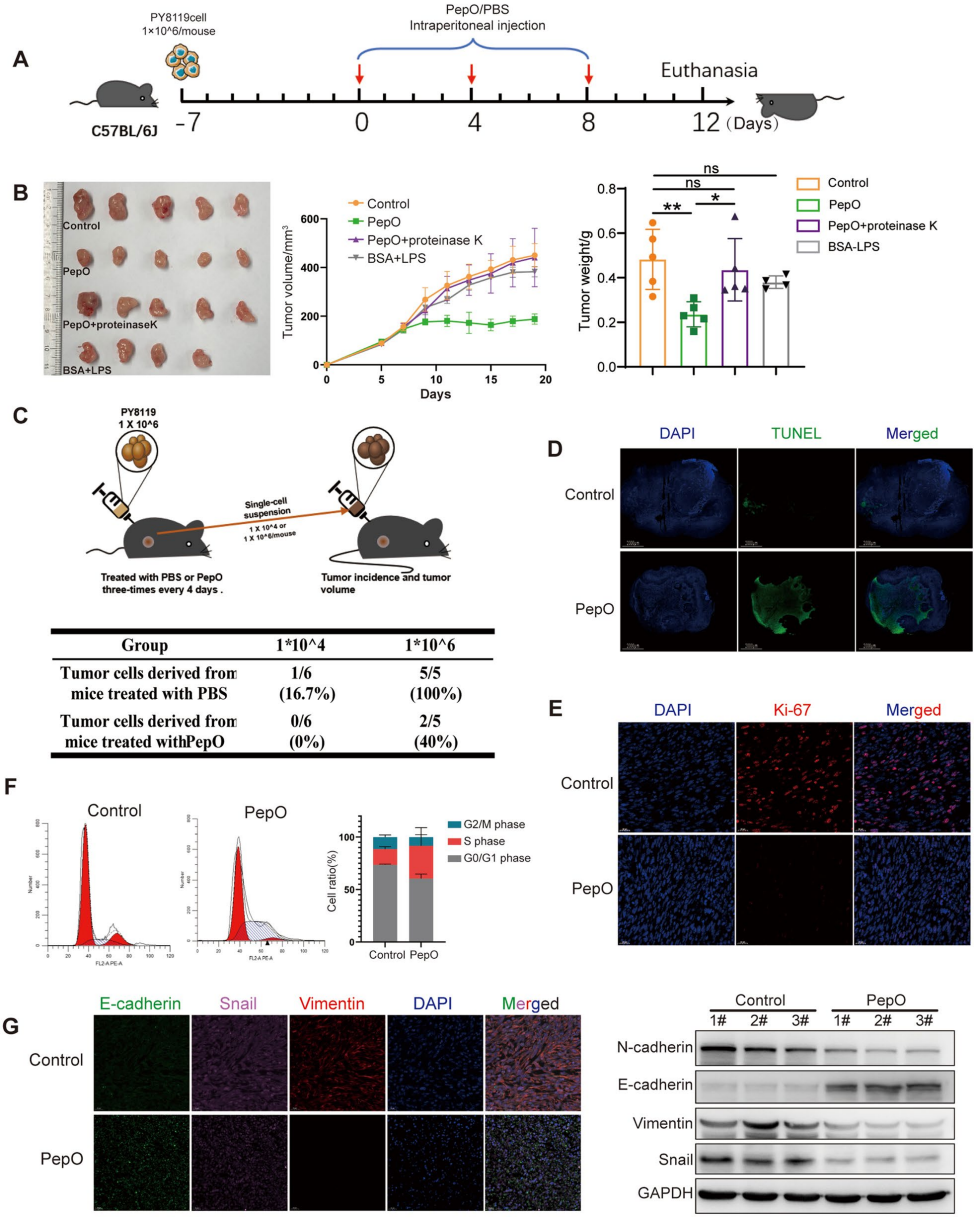
PepO treatment suppresses TNBC growth in vivo. **A** Schematic of the TNBC model and PepO/PBS treatment regimen. At the end of the experiments, mice were sacrificed, and the anticancer effects in each group were evaluated and compared (n = 5). **B** Tumor growth profiles in different treatment groups, and the tumor weights at the end of the experiment was recorded (n = 5). **C** Schematic of the secondary tumorigenesis and statistics table of tumorigenicity. **D**, **E** Tumor tissues were excised, fixed and sectioned. TUNEL staining of tumor tissues from each group was used to evaluate the apoptosis of tumor cells (scale bar = 1000 μm). Cell proliferation (Ki-67) was evaluated using Immunofluorescence detection (scale bar = 20 μm). **F** Flow cytometry was used to analize the phase of the tumor cell cycle from tumor tissue treated by PepO or PBS congtrol. The mean cell ratios at every phases were shown in the right graph. **G** Left, immunofluorescent triple staining for E-cadherin (green), Snail (pink), Vimentin (red) in TNBC tissue established by PY8119. Right, western blot detection of EMT markers in tumor tissue treated or untreated by PepO. Two-way repeated-measures ANOVA with Sidak’s multiple comparisons test was used in (B, Tumor growth profiles), and one-way ANOVA with Tukey’s multiple comparisons test was used in (B, tumor weights). Bar graphs represent mean ± SEM, *P < 0.05, **P < 0.01

The previous data indicated that PepO may plays an anti-tumor role in vivo. We further verified this hypothesis using a PY8119 TNBC mouse model (Fig. 2A). Mice treated with PepO specifically and more efficiently reduced tumor volumes and growth rates in comparation with the negative control (PBS), BSA + LPS (equivalent dose of LPS to exclude the interference of LPS) and PepO-proteinase treatment groups (Fig. 2B). The tumor-suppressive effect of PepO was also dose-dependent (Additional file 1: Fig S2A), given the anti-TNBC effect of PepO was greater at 1 mg/mouse, we used this dose of PepO for the subsequent experiments in vivo. We further evaluated the long-term effect of PepO on the proliferation and tumorigenicity of tumor cells in vivo*.* PY8119 cells from tumors of 1st TNBC-bearing mice were isolated and transplanted into the 2nd new healthy mice (Fig. 2C upper). And the result showed that after PepO treatment, PY8119 cells lost nearly all tumorigenic properties and the levels of tumor formation were reduced to 16.7% and 60% using 1 × 10^4^ and 1 × 10^6^ cells/mouse i.p., respectively (Fig. 2C lower and Additional file 1: Fig S2B). Additionally, PepO treatment also resulted in a decrease in the tumor stem cell-related proteins ABCG2, OCT4, SOX2 and Olig2 (Additional file 1: Fig S2C).

We further explored the anti-tumor mechanism of PepO administration and assayed apoptosis, proliferation, cell cycle and epithelial-mesenchymal transition (EMT) markers directly from tumor tissues. PepO administration resulted in higher levels of apoptotic cells in the tumors than the negative controls using TUNEL staining (Fig. 2D). PepO treatment also suppressed PY8119 cell proliferation (Fig. 2E) and prolonged their S phase (Fig. 2F).

EMT increases cancer cell invasiveness as well as the numbers of cancer stem cells, circulating tumor cells and encourages drug resistance [32]. We found that PepO had reversed the EMT process by increasing the levels of the epithelial protein E-cadherin and decreasing the mesenchymal proteins Snail and Vimentin (Fig. 2G, left). These results were mirrored in Western blots where PepO upregulated the E-cadherin protein levels and downregulated the mesenchymal-associated N-cadherin, Vimentin and Snail compared with controls in the tumor tissues (Fig. 2G, right). We also evaluated tumor cell migration using the in vitro scratch wound healing assay. PepO added to the cells reduced the rate of cell migration to heal the disrupted line of cells (Additional file 1: Fig S2D). These data suggested that PepO acted to reverse the EMT process and inhibit the migration and invasion of PY8119 cells.

We next measured blood biochemical markers of health in our experimental mice to detect potential adverse effects of PepO treatment. Importantly, the high dose of PepO we used for our in vivo experiments did not result in elevated levels of liver, heart or kidney damage i.e., albumin (ALB), alkaline phosphatase (ALKP), alanine aminotransferase (ALT), aspartate aminotransferase (AST), creatinine (CREA), urea and creatine kinase (CK) were all normal compared with controls (Additional file 1: Fig S2E). In addition, the body weights of the mice were not altered by PepO (Additional file 1: Fig S2F). Histological evaluations of heart, liver, spleen, lung, kidney, and bone marrow from PepO-treated mice revealed no signs of toxicity compared with controls (Additional file 1: Fig S2G). Together, these results indicated that PepO is a safe immunomodulator and possesses a significant anti-tumor effect.

### *PepO reprograms TAMs toward tumoricidal M1 *in vivo

**Fig 3:**
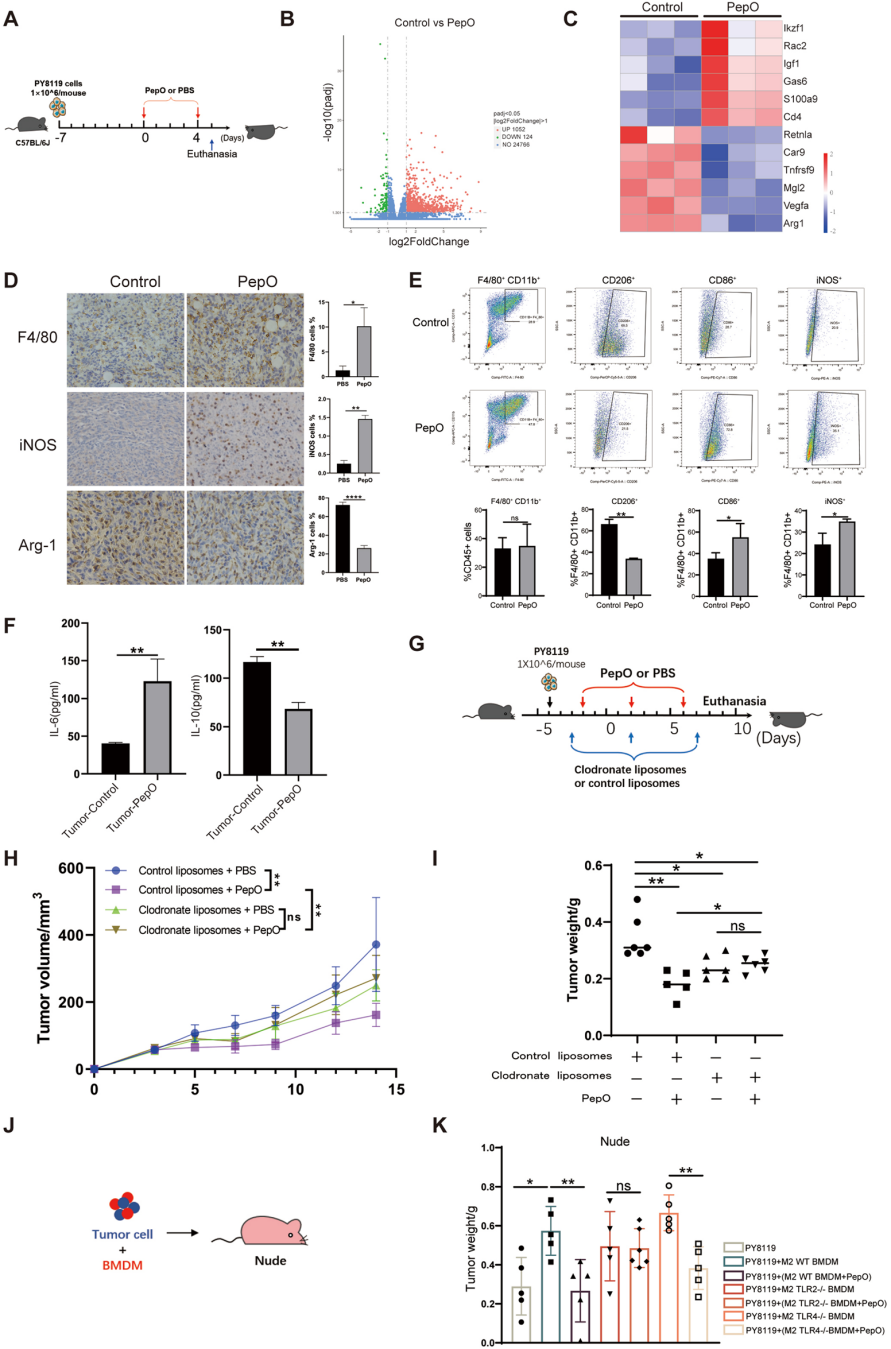
PepO reprograms TAMs toward tumoricidal M1 in vivo. **A** Schematic of the TNBC model and PepO/PBS treatment regimen. 24 h after the second injection of PepO or PBS, mice were sacrificed, and the tumor tissue were collected (n = 3). **B** Volcano plot of DEGs in tumor tissue treated by PBS or PepO. The red and green dots represent significantly up-regulated and down-regulated genes, respectively. **C** Heatmap of key DEGs in groups including PBS control and PepO treated. **D** IHC staining of F4/80, iNOS and Arg-1 analysis and quantification of the positive cells in all field (n = 3, scale bar = 20 μm). **E** FACS dot plots showing % M2 macrophages (CD45^+^F4/80^+^CD11b^+^CD206^+^) and % M1 macrophages (CD45^+^F4/80^+^CD11b^+^CD86^+^ or CD45^+^F4/80^+^CD11b^+^iNOS^+^) derived from tumor tissue of TNBC bearing mice treated by PepO or not (n = 3). **F** The pro-inflammatory cytokine IL-6, and anti-inflammatory cytokine IL-10 in tumor tissue were detected using ELISA kit (n = 3). **G** Schematic of the TNBC model and the injection of clodronate/control liposomes through tail vein 24 h before treatments of PBS or PepO (n = 6). **H**, **I** Tumor growth profiles and tumor weight were detected in TNBC-bearing C57BL/6 mice treated with control liposomes + PBS, control liposomes + PepO, clodronate liposomes + PBS,clodronate liposomes + PepO, respectively (n = 6). **J**, **K** 1 × 10^5 PY8119 cells or an admixture of 1 × 10^5 PY8119 cells and 1 × 10^6 BMDMs treated as shown were injected into nude mice (n = 5 each). The tumors were collected at 4 weeks after injection and the tumor weights were compared. Two-tailed Student t test was used in (**D**, **E**, **F**). Two-way repeated-measures ANOVA with Sidak’s multiple comparisons test was used in (**H**). One-way ANOVA with Tukey’s multiple comparisons test was used in (**I**, **K**). Bar graphs represent mean ± SEM, *P < 0.05, **P < 0.01

Given that PepO can reprogram macrophages to be tumoricidal in vitro and that PepO suppressed TNBC in vivo, we explored the role of PepO in suppressing tumor growth in vivo. We first focused on changes to the TME induced during early phases of PepO treatment. PY8119 cells were used to establish the TNBC model and tumors were collected 24 h following the second intraperitoneal injection of PepO or PBS (Fig. 3A). RNA-seq of the bulk tumors was used to explore transcriptome alterations that were regulated by PepO. We found 1052 upregulated genes in the PepO treatment and 124 in the PBS controls (Fig. 3B). In particular, the T cell activation genes Rac2 [33] and Igf1 [34] were increased while the malignant tumor-associated genes Mgl2 [35], Tnfrsf9 [36], Vegfa [37] as well as the M2 macrophage markers Retnla and Arg-1 were decreased (Fig. 3C). These data indicated that PepO attenuated the malignancy of the tumors and inhibited formation of the M2 phenotype. We performed further experiments to verify that M2 to M1 polarization had occurred within the tumors using both immunohistochemistry and multicolor immunofluorescence. PepO treatment dramatically increased tumor infiltrating macrophages (F4/80^+^) and iNOS^+^ M1 macrophages while decreasing Arg-1^+^ M2 macrophages in TME (Fig. 3D and Additional file 1: Fig S3A). Flow cytometry analysis (refer to Additional file 1: Fig S3B for gating strategies) also revealed that PepO treatment altered the overall composition of the TAM. In particular, the CD206^+^ M2 predominated in the tumors of the PBS treatment group (69.5 vs 21.5%) while the CD86^+^ M1 predominated in PepO-treated mice (72.8 vs 28.7%) (Fig. 3E). These data confirmed the effect of PepO on polarizing M2 TAMs toward M1 in vivo. Further evidence of this switch was provided by elevated iNOS + macrophages from 20.9% to 35.1% in the PepO-treated group and a robust response of the increased pro-inflammatory cytokine IL-6 and decreased anti-inflammatory IL-10 levels in crude lysate of tumors (Fig. 3F).

Those results suggested that PepO acts to reprogram TAM into tumoricidal M1 macrophages in vivo. However, whether PepO anti-tumor effect was dependent on macrophages remained to be clarified. We therefore depleted macrophages of PY8119-bearing mice using clodronate liposomes (Fig. 3G) and monitored the efficiency of macrophage depletion in blood and bone marrow (Additional file 1: Fig S3C). Macrophage depletion effectively disrupted the inhibitory effect of PepO on tumor growth and there was a further indication that the anti-tumor effect of PepO was macrophage-dependent (Fig. 3H, I). Macrophage depletion also resulted in a failure of PepO to maintain the tumor cells in S phase (Additional file 1: Fig S3D). Taken together, these data clearly demonstrated that PepO suppressed tumor growth in a macrophage-dependent manner.

We further verified the role for PepO-primed macrophages using PepO- and PBS-primed M2 BMDM mixed with PY8119 cells and transplants into nude mice (Fig. 3J). Tumor growth was promoted when PY8119 cells were co-cultured with M2 BMDM while PepO-primed M2 BMDM restricted tumor cell growth (Fig. 3K and Additional file 1: Fig S3E). In a similar manner, PepO-primed M2 cells reduced M2-driven EMT (Additional file 1: Fig S3F). Collectively, PepO reprogramed the tumor microenvironment into an anti-tumor M1 microenvironment and decreased the tumor-promoting properties of M2 macrophages.

### PepO reprogrammed M2 macrophage to be tumoricidal M1 by activating PI3K-AKT-mTOR and inhibiting JAK2-STAT3 pathway

**Fig. 4:**
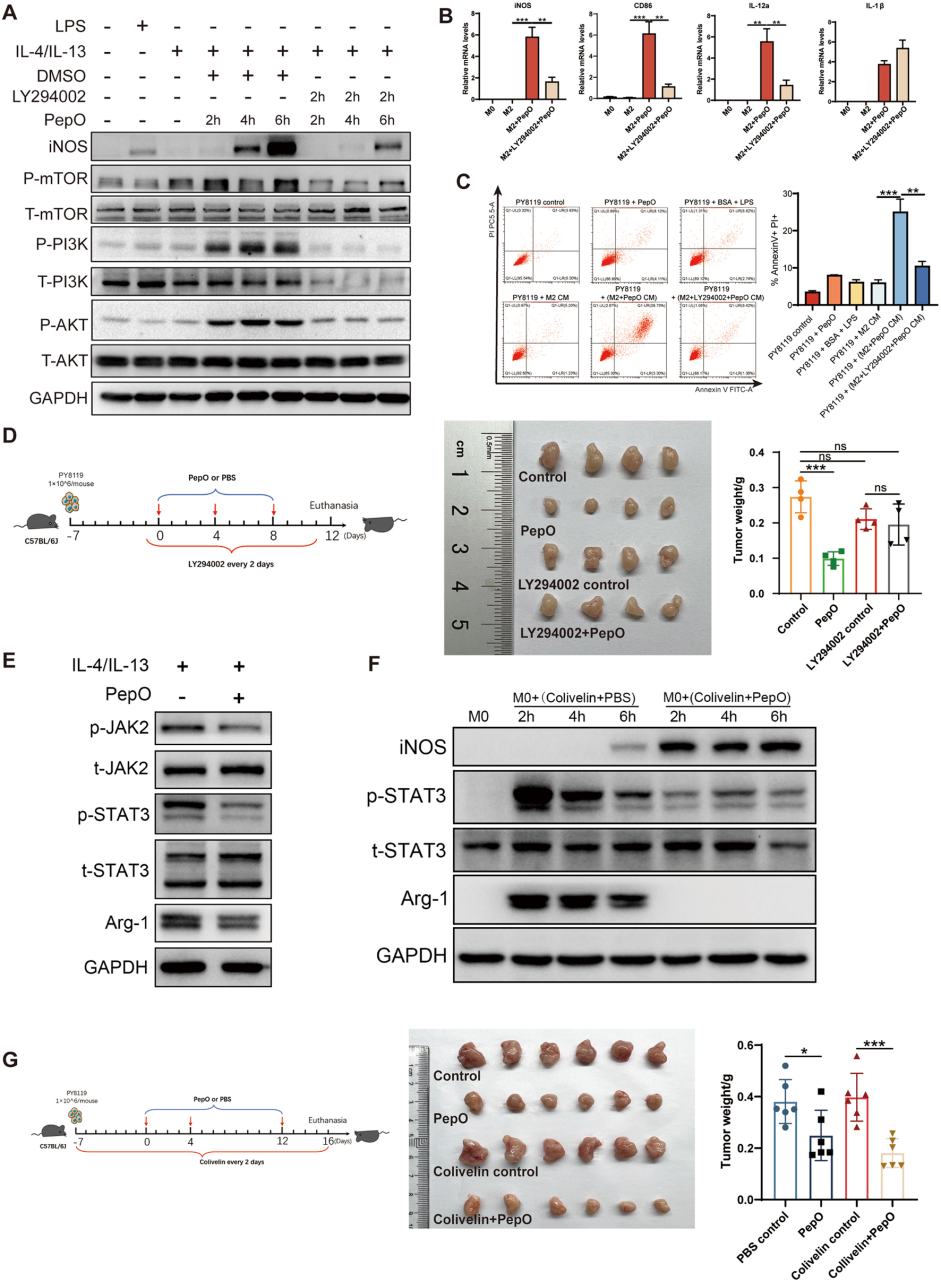
PepO reprogrammed M2 macrophage to be tumoricidal M1 by activating PI3K-AKT-mTOR and inhibiting JAK2-STAT3 pathway. **A** BMDM was treated as shown and the phosphorylation of PI3K-AKT-mTOR and the expression of iNOS was evaluated by western blot, 50 μM of LY294002 was used to block PI3K pathway 2 h before PepO treatment. **B** M2 BMDM pretreated with LY294002(50 μM) for 2 h, and then add PepO(5ug/ml) to the medium for 24 h. The transcription level of M1 markers were measured by RT-qPCR (n = 3). **C** FACS analysis of annexin V/PI staining and quantification of PY8119 cells treated with indicated CM for 48 h (n = 3). **D** PY8119 cells were used to establish TNBC in vivo and LY294002(50 mg/kg) was used to inhibit PI3K signaling pathway 24 h before PepO injection, and LY294002 was injected through i.p. every 2 days (D, left). Tumors were collected and the tumor growth was assessed by weight (n = 4, D, right). **E** BMDM were polarized into M2 by IL-4 and IL-13 for 24 h, and treated with PepO (5ug/ml) for another 24 h. The phosphorylation of JAK2-STAT3 and the expression of Arg-1 was evaluated by western blot. **F** BMDM was treated with colivelin (50 μg/ml) at the presence of PepO or PBS and the phosphorylation of STAT3 and the expression of iNOS and Arg-1 was evaluated by western blot. **G** PY8119 cells were used to establish TNBC in vivo and colivelin(1 mg/kg) was used to activate STAT3 signaling pathway 24 h before PY8119 injection, and colivelin was injected through i.p. every 2 days (F, left). Tu mors were collected and the tumor growth was assessed by weight (n = 6, F, right). One-way ANOVA with Tukey’s multiple comparisons test was used in (B), (C, right), (D, right), and (G, right). Bar graphs represent mean ± SEM, *P < 0.05, **P < 0.01, ***P < 0.001

Next, we wondered that how PepO reprogrammed TAMs to be tumoricidal M1 macrophage. It had been reported that macrophage polarized to M1 phenotype when PI3K-AKT pathway was activated [38]. Hence, we verified whether PI3K-AKT pathway was activated when M2 macrophage was polarized into M1 by PepO. And the PI3 kinase inhibitor LY294002 was administrated to evaluate the requirement of PI3K-AKT pathway involved in macrophage polarization. We found that PepO activated PI3K-AKT pathway by upregulating phosphorylation of PI3K, AKT and mTOR and then promote the expression of iNOS in a time-dependent manner. And LY294002 would abrogate the activation by PepO (Fig. 4A). Likewise, PepO treatment upregulated M1 related genes iNOS, CD86, IL-12a, and LY294002 neutralized this upregulation (Fig. 4B). However, the administration of LY294002 did not influence the inhibition of M2 phenotype by PepO (Additional file 1: Fig S4A). Subsequently, we found that PepO treatment could lead to PY8119 cell apoptosis (28.73%) while the administration of LY294002 reduced the apoptosis cells (9.42%) (Fig. 4C). Likewise, in TNBC-bearing mice, PepO significantly suppressed tumor growth, while LY294002 abrogated the anti-tumor property of PepO (Fig. 4D). These results suggested that PepO conferred M2 macrophages M1 property by activating PI3K-AKT-mTOR pathway and inhibited tumor growth in vivo.

Numerous studies had reported that the activation of JAK2-STAT3 pathway was involved in M2 formation [39, 40]. We found that PepO significantly inhibited the phosphorylation of JAK2-STAT3 pathway and downregulated Arg-1 (Fig. 4E). To further confirm that PepO prevents TAM from M2 phenotype through JAK2-STAT3, we employed STAT3 agonist colivelin to verify this. According to the immunoblot, colivelin activated STAT3 of M0 BMDM and M0 BMDM was polarized into M2 phenotype by elevating Arg-1, while PepO could suppress the STAT3 phosphorylation and reversed M2 macrophages to M1 phenotype at the presence of colivelin (Fig. 4F), suggesting that PepO indeed prevented macrophages from M2 by inhibiting JAK2-STAT3 pathway. Moreover, to further investigate whether PepO inhibit M2 macrophage and tumor growth by inhibiting the JAK2-STAT3 pathway in vivo, colivelin was used to activate STAT3 in TNBC-bearing mice. PepO as always suppressed cancer growth, and PepO still suppressed tumor growth at the presence of colivelin (Fig. 4G), which was consistent with the result of immunoblot (Fig. 4F), which suggested that PepO may inhibit a certain molecular upstream of STAT3.

Collectively, PepO reprogramed tumor-promoting M2 to tumor-inhibitory M1 by activating PI3K-AKT and inhibiting JAK2-STAT3 pathway, and suppressed TNBC.

### *PepO reprogramed M2 macrophages *via* TLR2 and TLR4 recognition*

**Fig. 5:**
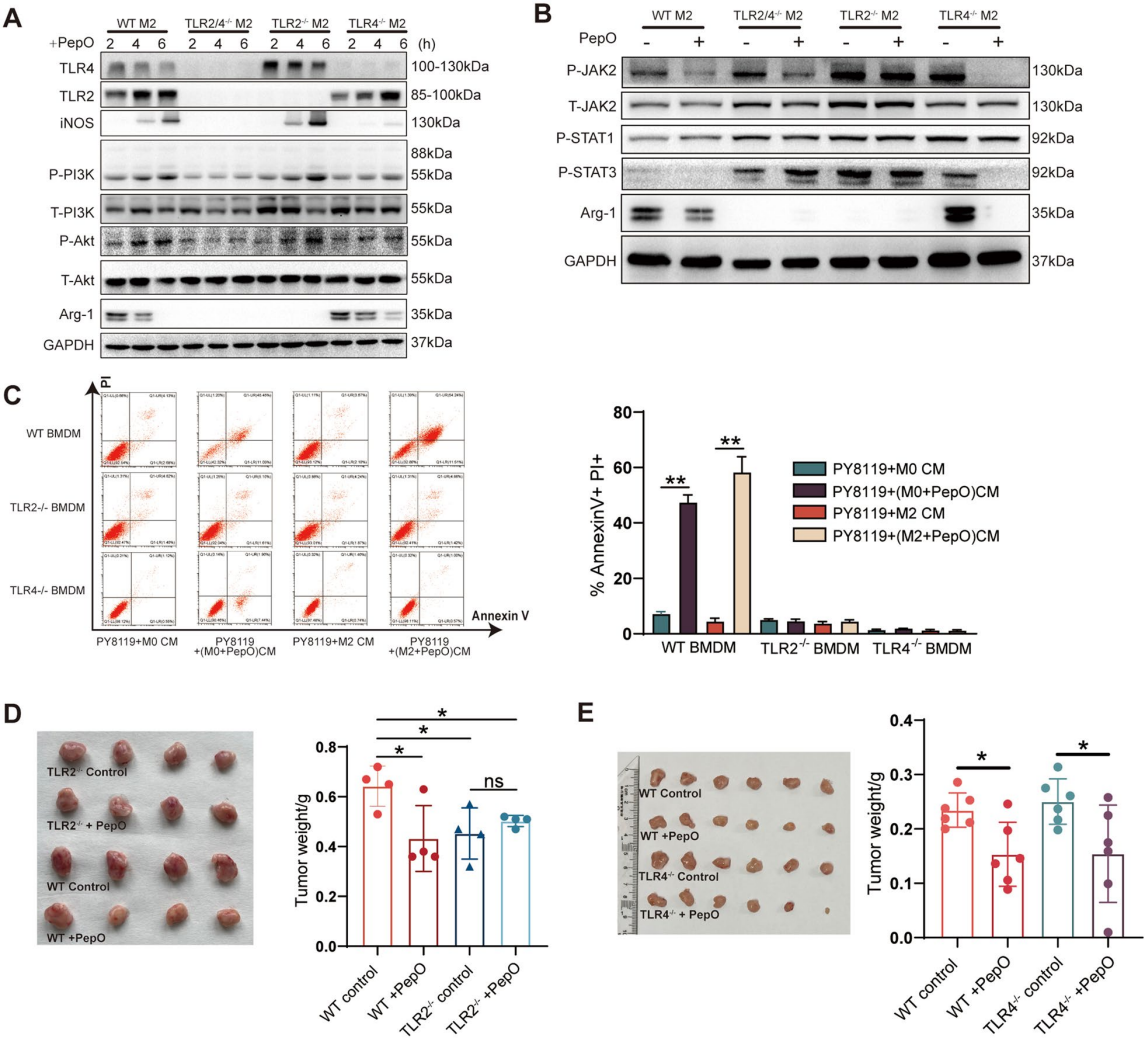
PepO reprogramed M2 macrophages via TLR2 and TLR4 recognition. **A** WT and TLR2 and/or TLR4 M2 BMDMs were stimulated with PepO(5 μg/ml), and samples were collected 2, 4, 6 h after stimulation. The phosphorylation of PI3K-AKT pathway associated proteins and M1 and M2 markers were evaluated by western blot. **B** WT and TLR2 and/or TLR4 M2 BMDMs were stimulated with PepO(5 μg/ml) for 24 h, The phosphorylation of JAK2-STAT3 pathway and the expression of Arg-1 was evaluated by western blot. **C** PY8119 cells were co-cultured with indicated CM for 48 h, and the percentage of apoptotic PY8119 cells labeled with PI and Annexin V were detected by FACS (n = 3). **D**, **E** TNBC was established in TLR2-/- (D, n = 4) and TLR4-/- (E, n = 6). Tumors were collected as previous schedule and the weight of tumor was measured. Two-way ANOVA with Tukey’s multiple comparisons test was used in (C). One-way ANOVA with Tukey’s multiple comparisons test was used in (**D**, **E**). Bar graphs represent mean ± SEM, *P < 0.05, **P < 0.01

Our previous study indicated that PepO induced the innate immune response in a TLR2/TLR4-dependent manner [26, 31]. We used BMDM derived from TLR2- and/or TLR4-deficient mice to test whether the switch of M2 macrophages to M1 was dependent on either TLR2 or TLR4. Interestingly, PepO was not able to activate the PI3K-AKT pathway of M2 macrophages and not able to increase iNOS mRNA or protein expression in TLR4-deficient M2 BMDM and did not increase mRNA levels of CD86, IL-1β and IL-12a (Fig. 5A and Additional file 1: Fig S5A), which indicated that PepO could not switch TLR4^−/−^ M2 BMDM toward the anti-tumor M1 phenotype. In contrast, PepO inhibited M2 polarization by inhibiting the activation of JAK2-STAT3 pathway and reducing the expression of Arg-1 and the mRNA levels of Arg-1, Fizz-1, CD206 and Ym1 in TLR4^−/−^ BMDM (Fig. 5B and Additional file 1: Fig S5A). Similar experiments using TLR2^−/−^ BMDM resulted in a time-dependent increase in phosphorylation of PI3K-AKT pathway and iNOS and increased mRNA levels of the M1 markers iNOS, CD86, IL-1β and IL-12a (Fig. 5A and Additional file 1: Fig S5A). In contrast, the TLR2^−/−^ BMDM were not able to be polarized into M2 macrophages by the administration of IL-4 and IL-13 to the cultures, which was similar to Chang’s findings [41]. And PepO had no effect on the phosphorylation of JAK2-STAT3 pathway in TLR2^−/−^ BMDM (Fig. 5B). Additionally, PepO could induce neither M2 nor M1 macrophage phenotypes in TLR2/4^−/−^ BMDM (Fig. 5A, B and Additional file 1: Fig S5A). These data indicated that on the one hand, PepO provoked M2 to polarize toward anti-tumor M1 phenotype via TLR4 recognition and activation of PI3K-AKT pathway. On the other hand, PepO inhibited M2 polarization via TLR2 recognition and inhibition of JAK2-STAT3 pathway.

We next explored whether the absence of either TLR2 or TLR4 affected the anti-tumor properties of PepO in vitro and in vivo. The apoptosis of PY8119 cells was eliminated using PepO-primed TLR2^−/−^or TLR4^−/−^ BMDM compared with wildtype BMDM in vitro (Fig. 5C). Then we also evaluated the anti-tumor property of PepO in TLR2^−/−^ and TLR4^−/−^ mice. In TLR2 deficiency groups, PepO failed to suppress tumor growth (Fig. 5D), which was consistent with cell apoptosis in vitro. However, compared the TLR2^−/−^ control group with the wildtype control group, TLR2 deficient exerted an anti-tumor effect without PepO (Fig. 5D). Western blotting also indicated that TLR2^−/−^ BMDM could not be reprogramed into M2 macrophages [41] (Fig. 5A, B). Previous studies have indicated that TLR2 deficiency or the presence of TLR2 antagonists can effectively inhibit tumor growth [42]. This is consistent with the slow tumor growth we found in the TLR2^−/−^ mice. In addition, the anti-tumor effect of PepO-primed M0 macrophages was not as strong as PepO-primed M2 macrophages (Fig. 1E and Additional file 1: Fig S1C). Therefore, in our model, although PepO could induce TLR2^−/−^ BMDM to express iNOS, TLR2 deficiency could also inhibit tumor growth and partially concealed the tumor-killing effect of PepO-primed M2 macrophages. Nevertheless, in the TLR4^−/−^ mice, PepO treatment effectively inhibited tumor growth (Fig. 5E) via preventing TAM from polarizing into M2 tumor-promoting phenotype.

We further pursued the mechanism of this phenomenon and used immunofluorescence assays to monitor tumor cell proliferation and EMT in vivo. Tumor cell proliferation in TLR2^−/−^ mice was not promoted because they lacked M2 macrophages while TLR4^−/−^ mice displayed a reduced tumor cell proliferation in response to PepO treatment owing to PepO could prevent TLR4 deficient macrophages from tumor-promoting M2 phenotype (Additional file 1: Fig S5B). The cancer cells in TLR2^−/−^ mice were remained epithelioid phenotype while in TLR4^−/−^ mice, PepO could effectively inhibit the EMT (Additional file 1: Fig S5C). Therefore, the anti-tumor effect of PepO in TLR2^−/−^ mice was partly concealed by TLR2 deficiency, while in TLR4^−/−^mice, although PepO cannot induce TAM (M2) to polarize into iNOS^+^ M1 like macrophages, PepO significantly inhibited TAM polarizing to the M2 phenotype (Fig. 5A, B), which reduced the tumor promoting effect of the M2 cells and ultimately inhibited tumor growth.

We further verified this by mixing PY8119 cells with PepO- or PBS-primed TLR2^−/−^ BMDM or TLR4^−/−^ BMDM and then the mixture was injected into nude mice. TLR2^−/−^ macrophages slightly inhibited tumor growth and PepO-priming did not significantly alter this inhibition. However, PepO-primed TLR4^−/−^ M2 BMDM effectively inhibited tumor growth (Fig. 3K). These results were consistent with the results in TLR2 and TLR4 deficient mice (Fig. 5D, E). Likewise, PepO-primed TLR4^−/−^ M2 BMDM effectively inhibited tumor cell proliferation and TLR2^−/−^ TAM could not promote tumor cell proliferation and reverse EMT due to the lack of M2 macrophages. In contrast, PepO-primed TLR4^−/−^ M2 BMDM reversed the EMT process (Additional file 1: Fig S5D, E). These results were also consistent with those in the TLR2- and TLR4-deficient mice. The proliferation of PY8119 cells in vitro was not enhanced by TLR2^−/−^ M2 BMDM and could not be inhibited by PepO-primed TLR2^−/−^ M2 BMDM, while TLR4^−/−^ M2 BMDM promoted tumor cell proliferation and this could be inhibited by PepO reprograming (Additional file 1: Fig S5F). Collectively, PepO provoked M2 macrophages to polarize toward the anti-tumor M1 phenotype, which was dependent on both TLR2 and TLR4.

### PepO enhances the anti-TNBC effect in combination with doxorubicin

**Fig. 6:**
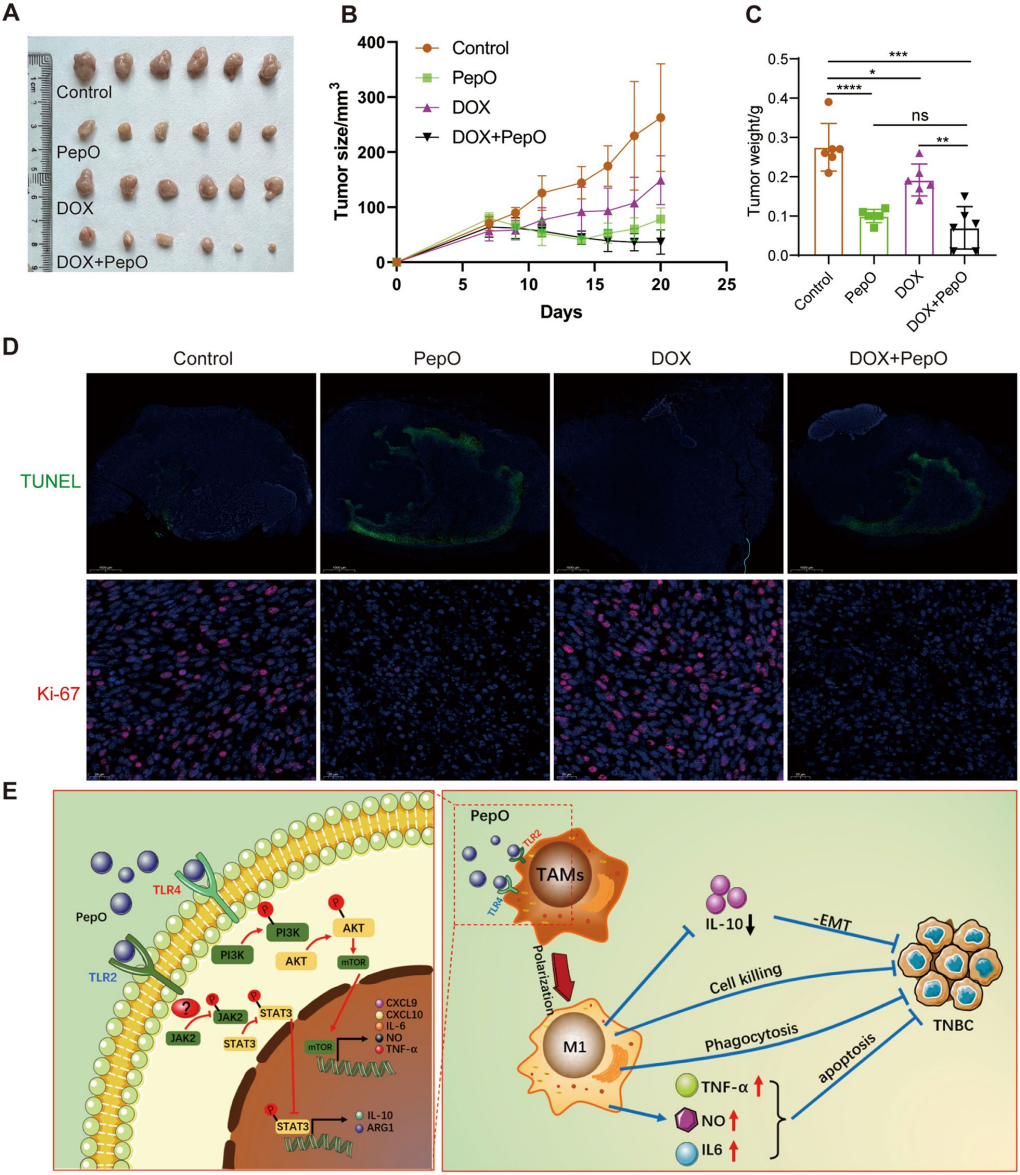
PepO enhances the anti-TNBC effect in combination with doxorubicin. **A**–**C** PY8119 bearing C57BL/6 mice were treated with PBS, PepO, doxorubicin (5 mg/kg), and the combination. The images of tumer (**A**), the tumor growth profiles (**B**) and tumor weight (**C**) were collected as shown (n = 6). **D** TUNEL staining of tumor tissues from each group was used to evaluate the apoptosis of tumor cells (scale bar = 1000 μm). Cell proliferation (Ki-67) was evaluated using Immunofluorescence detection (scale bar = 20 μm). **E** Model: PepO reprograms TAMs to anti-tumor M1 macrophages by activating PI3K-AKT-mTOR and inhibiting JAK2-STAT3 pathway via TLR2 / TLR4, consequently (1) Decrease the anti-inflammatory cytokines IL-10 and reverse the EMT process. (2) Enhance the functions of M1 macrophage related to cell killing, phagocytosis and NO biosynthetic process. (3) Increase the release of TNF-α, IL-6 and nitric oxide to promotes cancer cell apoptosis. Altogether, PepO suppresses the growth of TNBC. Two-way repeated-measures ANOVA with Sidak’s multiple comparisons test was used in (**B**). One-way ANOVA with Tukey’s multiple comparisons test was used in (**C**). Bar graphs represent mean ± SEM, *P < 0.05, **P < 0.01, ***P < 0.001, ****P < 0.0001

PepO suppresses tumor growth via activation of innate and adaptive immunity in vivo. We therefore examined whether PepO could increase the therapeutic action of doxorubicin [43]. We combined PepO with high-and low-dose doxorubicin to assess whether combination therapy could maximumly inhibit TNBC in the PY8119-bearing C57BL/6 mouse model. The high and low dosages of doxorubicin were less effective than PepO in suppressing tumor growth while combination therapy showed the maximum effect on tumor suppression (Fig. 6A–C and Additional file 1: Fig S6A–C). This indicated that PepO significantly enhanced the drug sensitivity of the tumor cells. In addition, doxorubicin did not interfere with the PepO functions of increasing tumor tissue apoptosis and decreasing tumor cell proliferation (Fig. 6D). These results demonstrated that PepO combined with doxorubicin represented a better therapeutic effect.

## Discussion

Macrophage infiltration into breast cancer tissues is common in solid malignancies like TNBC and are associated with a poor clinical outcome [44]. In TNBC, the immunosuppressive microenvironment specificity can efficiently favor the polarization of macrophages toward M2, which dominate in the population of immune cells to exacerbate tumor progress. Thus, targeting tumor associated macrophages (TAM) is a promising strategy for cancer therapy. Several small molecule-based or antibody-based drugs have been created to deplete TAM populations and delay tumor progression in animal models [45, 46]. However, empirical investigations have also demonstrated that TAM reduction is not sufficient to provide long-lasting anticancer effects [47, 48]. TAM reprogramming is an alternative treatment strategy since plasticity and flexibility are essential characteristics of macrophages [49]. These reprogramming techniques provide the opportunity to actively enhance the antitumor immunological activity of TAM in addition to eliminating their tumor-supportive roles [50, 51]. However, how to drive reprogramming with high potency remains a challenge.

In this study, we proposed a strategy to reprogram the immune microenvironment of cancer. The *S. pneumoniae* endopeptidase O virulence protein PepO served as an immunoregulatory factor. The administration of PepO in vivo reversed immune suppression in the tumor microenvironment and induced a systemic antitumor response. We demonstrated that macrophages actually dominated the antitumor efficacy of PepO, and had provided clear evidence that PepO conducts antitumor effect through the immunoregulating function. We confirmed the anti-TNBC efficacy of PepO in TNBC-bearing mouse model. However, such antitumor effect was spoiled in macrophage-deficient condition, suggesting that PepO activated antitumor immunity in a macrophage- mediated manner. Moreover, PepO significantly switched TAM to the tumoricidal M1 macrophage as reflected by a reduction in the expression of M2 markers Arg-1, CD206, Fizz-1, Ym1 and IL-10 while increasing the expression of M1 markers iNOS, IL-12a and IL-1β and promoted apoptosis of the tumor cells. Given that macrophage gene expression patterns are often diverse and do not exactly match patterns associated with these M1/M2 classifications, we further analyzed the secretion of M1- and M2-related cytokines from PepO-primed M2 macrophage. PepO treatment substantially increased the secretion of M1-related cytokines including TNF-α and IL-6 and reduced the secretion of the M2-related cytokine IL-10. Next, we systematically analyzed altered genes of PepO-primed macrophages and showed that PepO switched M2 macrophages to M1 genetically and enhanced the functions of macrophage related to cell killing, phagocytosis and nitric oxide biosynthetic process.

Several signaling pathways are involved in macrophage polarization including Janus kinases/signal transduction and activator of transcription (JAK/STAT), Notch signaling pathway, PI3K/Akt [52–54] and some key transcription factors including nuclear factor-κB (NFκB), activator protein 1 (AP-1), peroxisome proliferator-activated receptor-γ (Pparγ), cAMP-responsive element-binding protein (CREB) and Krüppel-like factor 4 (KLF4) [55]. Our results demonstrated that PepO provoked M2 macrophages to polarize toward M1 by activating PI3K-AKT-mTOR and NFκB (data not shown) concomitant with the upregulation of M1-associated markers. The PI3K inhibitor abrogated the increase of M1 markers but did not affect the regression of M2 polarization primed by PepO, which indicated that PepO suppresses the M2 phenotype in another pathway, and then JAK2-STAT3 pathway was being monitored.

JAK2-STAT3 signal is associated with cell survival, angiogenesis, immunosuppression and tumor invasion in cancers [56, 57]. Murine studies have reported that STAT3 pathway in macrophages is involved in immune regulation [58]. The STAT3 pathway is more highly activated in response to several cytokines including IL-4 and IL-10 in TAMs of the M2 than in M1 phenotype [59]. Yue Zhong and his colleagues found that IL-10 stimulated the JAK2-STAT3 pathway in gloma cell line U271 and U87 through interaction with JAK2 in a protein–protein interaction fashion [60]. And Junyu Zhu had found that IL-10 expression was closely related to STAT3. So JAK2-STAT3 has a dual role in IL-10 mediated signaling. JAK2-STAT3 activation increase IL-10 expression, which in turn lead to JAK2-STAT3 phosphorylation, resulting in maintain macrophages M2 phenotype. However, in our research PepO-primed M2 macrophage downregulated IL-10 expression, which might break this circulation and reduced the phosphorylation of JAK2-STAT3 and leading to prevent TAM from M2 phenotype.

TLRs are innate immune system activators and play a role in inflammatory processes [61]. TLR signaling and macrophage polarization are tightly linked. Lung cancer cells can stimulate tumor metastasis by triggering macrophages via TLR2 signaling [62]. NO, Arg-1 and SCOS3 were induced in hepatoma-treated TLR2^−/−^ BMDM [41]. Paclitaxel (similar to LPS) induced M1 polarization that was abrogated in TLR4^−/−^ but not in TLR2^−/−^ BMDM [63], which was consistent with our results where TLR4 deficiency would abrogate PepO-driven M1 polarization while PepO still switched M2 to M1 macrophages in TLR2^−/−^ BMDM but TLR2^−/−^ BMDM could not be polarized toward M2. Classic literature reported that Toll-like receptor signaling promotes the production of inflammatory cytokines and thus promotes inflammation [64]. Nevertheless, evidence has suggested that TLR2 and TLR4 activation can either induce or put an end to the immune responses by controlling the activities of cells involved in both innate and adaptive immunity [65, 66]. TLR2 and/or TLR4 activation leads to the multitude of immune mediators production that engages receptors that could regulate JAK2 and the STAT3 transcription factor. JAK2-STAT3 activation results in inhibition of the functional dendritic cells maturation, increased T regulatory cell population and M1 to M2 macrophage polarization. Shao et al. found TLR4 phosphorylates PI3K-AKT signaling, accompanied by the activation of downstream pathways and the release of inflammatory factors [67]. Based on these findings, we had employed TLR2 and TLR4 deficient BMDM and mice to clarify the interaction beneath the PepO-driving macrophage polarization. Then we had made it clear that PepO, as a TLR2 and TLR4 dual ligand agonist, provoked M2 macrophage to M1 phenotype by activating PI3K-AKT pathway via TLR4, and prevent TAMs from M2 by inhibiting JAK2-STAT3 pathway via TLR2.

## Conclusion

In summary, our study demonstrated that PepO, as a high level of biosafe immunomodulatory molecule, switches TAM from the tumor-promoting M2 to tumor-inhibitory M1 phenotype by interacting with TLR2 and TLR4 and activating PI3K-AKT-mTOR and inhibiting JAK2-STAT3 pathway. PepO treatment enhanced the function of macrophage related to cell killing, phagocytosis and nitric oxide biosynthetic process and promoted the release of pro-inflammatory cytokines and NO that diffuse into adjacent tumor cells resulting in cell death, and leading to a decrease of anti-inflammatory cytokines IL-10 to reverse the EMT process. Additionally, PepO served as an effective sensitizer of the chemotherapy drug doxorubicin and the combined treatment contributed to the synergistic tumor-inhibitory effect. Therefore, PepO is a promising candidate drug for clinical TNBC immunotherapy (Fig. 6E).

### Supplementary Information

**Additional file 1: Figure S1.** PepO reprograms M2 macrophages towards M1 and to be tumoricidal. (A) Raw264.7 was co-cultured with PY8119 cells in the presence of PepO or not. The transcription level of M1 and M2 macrophage markers were measured using RT-qPCR. (B)The pro-inflammatory cytokine TNF-α and IL-6, and anti-inflammatory cytokine IL-10 in TAMs(Raw264.7) or TAMs treated by PepO were detected with ELISA kit. (C)The percentage of apoptotic PY8119 cells detected by FACS. (D) PY8119 cells were co-cultured with PBS, PepO control, and conditional medium (CM) of M0 BMDM or M0 BMDM treated by PepO, M2 BMDM or M2 BMDM treated by PepO, BMDM treated by PepO quivalents BSA and LPS. Then the apoptosis of 4T1 was assessed with annexin-V/PI kit and flow cytometry. (E) The expression of cleaved caspase-3(CC3) of PY8119 cells was evaluated via western blot. (F) RT-qPCR analysis was performed to examine the mRNA level of Cxcl9, Cxcl10 and Arg-1 in M2 phenotype BMDM and Raw264.7 treated by PepO or not. Two-way ANOVA with Tukey’s multiple comparisons test was used in (A)(n=3), and (F)(n=3). One-way ANOVA with Tukey’s multiple comparisons test was used in (B)(n=3) (C) and (D right)(n=3). Bar graphs represent mean ± SEM, *P<0.05,**P<0.01, ***P<0.001, ****P<0.0001. **Figure S2.** PepO inhibits TNBC growth in vivo. (A) Tumor growth profiles treated by high and low dose of PepO, and the tumor weights at the end of the experiment were recorded (n = 5). (B) The images of tumers of secondary tumorigenesis. (C) Western blot analysis of CSC marker expression in tumor tissue. (D) PY8119 cell migration was assessed with the woundhealing assay via co-culturing with conditioned medium. (E) Serum levels of ALT, AST, CREA, ALB, ALKP, CK of tumor-free C57BL/6 treated with PBS control or PepO(n=3). (F) Body weight of tumor-free C57BL/6 treated by PBS or PepO. (G) Representative H&E-stained sections of heart, liver, spleen, lung, kidney, or bone marrow. Serum and tissues were collected 4 days after the third treatment. Two-way repeated-measures ANOVA with Sidak’s multiple comparisons test was used in (A, Tumor growth profiles) and (F), and one-way ANOVA with Tukey’s multiple comparisons test was used in (A, tumor weights). Two-tailed Student t test was used in (E). Bar graphs represent mean ± SEM. **Figure S3.** PepO reprograms TAMs and TAMs acquire anti-tumor capability in vivo. (A)Immunofluorescent triple staining for macrophage markers F4/80 (red), Arg-1 (pink), iNOS (green) in TNBC tissue established by PY8119. (B) Gating strategy for flow cytometry of macrophage subsets (Isotype1 was stained with mouse APC-R700-labelled CD45 antibody and APC mouse IgG1 κ Isotype Ctrl and FITC mouse IgG1 κ Isotype Ctrl; Isotype2 was stained with mouse APC-R700-labelled CD45 antibody, mouse FITC-labelled F4/80 antibody, mouse APC-labelled CD11b antibody and perCP/cy5.5 Rat IgG2a κ Isotype, PE/Cyanine7 Rat IgG1, λ Isotype Ctrl and PE Rat IgG2b κ Isotype Ctrl). (C)The efficiency of macrophages depletion in blood, bone marrow. (D) Flow cytometry was used to analize the phase of the tumor cell cycle from tumor tissue grouped as shown. (E-F) Cell proliferation (Ki-67), and EMT markers of TNBC established in nude mice was evaluated (scale bar of Ki-67=200μm, scale bar of EMT markers=20μm). **Figure S4.** PepO reprogramed macrophages to be tumoricidal through activating PI3K-AKT- mTOR signaling pathway. (A) The transcription level of M2 markers were measured by RT-qPCR. One-way ANOVA with Tukey’s multiple comparisons test was used in (A). Bar graphs represent mean ± SEM, *P<0.05, **P<0.01, ***P<0.001. **Figure S5.** PepO reprogramed M2-like macrophage via being recognized by TLR2 and TLR4. (A)The transcription level of macrophage markers were determined by RT-qPCR in indicated groups shown in A. (B) Cell proliferation (Ki-67) was evaluated using Immunofluorescence detection(scale bar=20μm). (C) Immunofluorescent triple staining for E-cadherin (green), Snail (pink), Vimentin (red) in TNBC tissue grouped as shown (scale bar=20μm). (D-E) The cancer cell proliferation (D, scale bar=50μm) and EMT process(E, scale bar=50μm) of nude mice grouped as labeled was assessed and the representative images were shown. (F) PY8119 cell were co-cultured with PepO- or PBS-primed gene deficient M2 BMDM, and the proliferation of PY8119 cell were evaluated by Ki-67. One-way ANOVA with Tukey’s multiple comparisons test was used in (A). **Figure S6.** PepO combined with doxorubicin further inhibited tumor growth. (A-C) PY8119 bearing C57BL/6 mice were treated with PBS, PepO, doxorubicin (2mg/kg), and the combination. The images of tumer (A), the tumor growth profiles (B) and tumor weight (C) were collected as shown. Two-way repeated-measures ANOVA with Sidak’s multiple comparisons test was used in (B). One-way ANOVA with Tukey’s multiple comparisons test was used in (C). Bar graphs represent mean ± SEM, *P<0.05, **P<0.01, ***P<0.001.

**Additional file 2: Table S1.** Reagents used in this study. **Table S2.** Primer sequences used in RT-qPCR. **Table S3.** Antibody used in immunoblotting. **Table S4.** Anti-mouse antibodies used in flow cytometry.

## Data Availability

All data generated or analyzed during this study are included in this main text and its supplementary information files. In addition, all data from this study can be obtained from the corresponding author upon reasonable request.

## Abbreviations

TNBC: Triple negative breast cancer
TME: Tumor microenvironment
TAM: Tumor associated macrophage
M1: M1 phenotype macrophage
M2: M2 phenotype macrophage
PepO: Streptococcus pneumoniae endopeptidase O
PBS: Phosphate buffer solution
RT-qPCR: Real time quantitative PCR
ELISA: Enzyme linked immunosorbent assay
EMT: Epithelial-mesenchymal transition
TLR2: Toll-like receptor 2
TLR4: Toll-like receptor 4
ER: Estrogen receptor
PR: Progesterone receptor
HER2: Human epidermal growth factor receptor 2
iNOS: Inducible nitric oxide synthase
Arg-1: Arginase 1
NO: Nitric oxide
BMDM: Bone marrow derived macrophage
CM: Conditioned medium
TUNEL: TdT-mediated dUTP nick end labeling
ALB: Albumin
ALKP: Alkaline phosphatase
ALT: Alanine aminotransferase
AST: Aspartate aminotransferase
CREA: Creatinine
CK: Creatine kinase
