# Supplementary material for: The Streptococcus virulence protein PepO triggers anti-tumor immune responses by reprograming tumor-associated macrophages in a mouse triple negative breast cancer model
Source: Cell Biosci. 2023 Nov 4;13:198. doi: 10.1186/s13578-023-01153-w (PMC10625220; doi:10.1186/s13578-023-01153-w)
Supplement: Supplementary file 2 — Additional file 2: Table S1. Reagents used in this study. Table S2. Primer sequences used in RT-qPCR. Table S3. Antibody used in immunoblotting. Table S4. Anti-mouse antibodies used in flow cytometry. [file 13578_2023_1153_MOESM2_ESM.docx]

| Reagent name | |  | company |
| --- | --- | --- | --- |
| DMEM medium | |  | Gibco |
| Fetal bovine serum | |  | Ausbian/Aibco |
| Penicillin-Streptomycin(10000U/ml) | |  | Gibco |
| PeproTech m-CSF 10μg | |  | PeproTech |
| Recombinant Rhesus Macaque IL-4 | |  | Beyotime |
| Recombinant Rhesus Macaque IL-13 | |  | Beyotime |
| 0.25 Trypsin-EDTA 0.02% in HBSS | |  | BIOAGRIO |
| LY294002 | |  | Topscience |
| protease inhibitor cocktail | |  | Thermo Scientific |
| Phosphatase inhibitor cocktail | |  | Thermo Scientific |
| TRIzol | |  | Invitrogen |
| DnaseI | |  | Beyotime |
| IV collagenase | |  | BioFroxx |
| Mouse IL-6 ELISA MAX™ Deluxe | |  | biolegend |
| ELISA MAX™ Deluxe Set Mouse TNF-α | |  | biolegend |
| Mouse IL-10 ELISA MAX™ Deluxe |  | | biolegend |
|  |  | |  |

Table S1. Reagents used in this study

Table S2. Primer sequences used in RT-qPCR

| Gene name | Primer sequence |
| --- | --- |
| iNOS | Forward:5’ GTTCTCAGCCCAACAATACAAGA 3’ |
|  | Reverse:5’ GTGGACGGGTCGATGTCAC 3’ |
| Arg-1 | Forward:5’ CTCCAAGCCAAAGTCCTTAGAG 3’ |
|  | Reverse:5’ GGAGCTGTCATTAGGGACATCA 3’ |
| Fizz-1 | Forward:5’ CCAATCCAGCTAACTATCCCTCC 3’ |
|  | Reverse:5’ ACCCAGTAGCAGTCATCCCA 3’ |
| Ym1 | Forward:5’ CAGGTCTGGCAATTCTTCTGAA 3’ |
|  | Reverse:5’ GTCTTGCTCATGTGTGTAAGTGA 3’ |
| CD206 | Forward:5’ GTGGAGTGATGGAACCCCAG 3’ |
|  | Reverse:5’ CTGTCCGCCCAGTATCCATC 3’ |
| IL-10 | Forward:5’ CAGTACAGCCGGGAAGACAA 3’ |
|  | Reverse:5’ CCTGGGGCATCACTTCTACC 3’ |
| IRF5 | Forward:5’ GGTCAACGGGGAAAAGAAACT 3’ |
|  | Reverse:5’ CATCCACCCCTTCAGTGTACT 3’ |
| CD86 | Forward:5’ CTGGACTCTACGACTTCACAATG 3’ |
|  | Reverse:5’ AGTTGGCGATCACTGACAGTT 3’ |
| IL-12a | Forward:5’ CTCTTTTTGGCCACCCTTGC 3’ |
|  | Reverse:5’ TCTTCAGCAGGTTTCGGGAC 3’ |
| IL-1β | Forward:5’ ATGAAAGACGGCACACCCAC 3’ |
|  | Reverse:5’ GCTTGTGCTCTGCTTGTGAG 3’ |
| Ccl3 | Forward:5’ ACTGCCTGCTGCTTCTCCTACA 3’ |
|  | Reverse:5’ AGGAAAATGACACCTGGCTGG 3’ |
| Cxcl9 | Forward:5’ CCGAGGCACGATCCACTACA 3’ |
|  | Reverse:5’ AGTCCGGATCTAGGCAGGTTTG 3’ |
| Cxcl10 | Forward:5’ ATCCGGAATCTAAGACCATCAAGAA 3’ |
|  | Reverse:5’ GGACTAGCCATCCACTGGGTAAAG 3’ |
| GAPDH | Forward:5’ TGGCCTTCCGTGTTCCTAC 3’ |
|  | Reverse:5’ GAGTTGCTGTTGAAGTCGCA 3’ |

Table S3. Antibody used in immunoblotting

| Antibody name | Company |
| --- | --- |
| Anti-GAPDH rabbit pAb | Abcam |
| Anti-iNOS rabbit mAb | Cell Signaling Technology |
| Anti-Arg-1 mouse mAb | Santa Cruz |
| Anti-E-cadherin mouse mAb | Abcam |
| Anti-snail mouse mAb | Cell Signaling Technology |
| Anti-Vimentin mouse mAb | Abcam |
| Anti-phospho-PI3K mAb | Cell Signaling Technology |
| Anti-PI3K mAb | Cell Signaling Technology |
| Anti-mTOR mAb | Cell Signaling Technology |
| Anti-phospho-mTOR mAb | Cell Signaling Technology |
| Anti-phospho-AKT mAb | Cell Signaling Technology |
| Anti-AKT mAb | Cell Signaling Technology |
| p-JAK2 | Cell Signaling Technology |
| t-JAK2 | Cell Signaling Technology |
| p-STAT1 | Cell Signaling Technology |
| p-STAT3 | Cell Signaling Technology |
| t-STAT3 | Cell Signaling Technology |
| Anti-TLR4 pAb | Cell Signaling Technology |
| Cleaved Caspase-3 | Abcam |

| Antibody name | Company |
| --- | --- |
| Ms CD16/32 Pure 2.4G2 | BD |
| Mouse APC-R700-labelled CD45 antibody | Biolegend |
| Mouse FITC-labelled F4/80 antibody | Biolegend |
| Mouse APC -labelled CD11b antibody | Biolegend |
| Mouse PE-cy7-labelled CD86 antibody | Biolegend |
| Mouse percp/cyanine5.5-labelled CD206 antibody | Biolegend |
| Mouse PE-labelled iNOS antibody | Biolegend |
| Mouse Brilliant Violet 421™ -labelled CD3 antibody | Biolegend |
| APC Mouse IgG1, κ Isotype Ctrl | Biolegend |
| FITC Mouse IgG1, κ Isotype Ctrl | Biolegend |
| PE/Cyanine7 Rat IgG1, λ Isotype Ctrl Antibody | Biolegend |
| PerCP/Cyanine5.5 Rat IgG2a, κ Isotype Ctrl | Biolegend |
| PE Rat IgG2b, κ Isotype Ctrl | Biolegend |
| Brilliant Violet 421™ Rat IgG2b, κ Isotype Ctrl | Biolegend |

Table S4. Anti-mouse antibodies used in flow cytometry
