# Supplementary material for: The Streptococcus virulence protein PepO triggers anti-tumor immune responses by reprograming tumor-associated macrophages in a mouse triple negative breast cancer model
Source: Cell Biosci. 2023 Nov 4;13:198. doi: 10.1186/s13578-023-01153-w (PMC10625220; doi:10.1186/s13578-023-01153-w)
Supplement: Supplementary file 1 — Additional file 1: Figure S1. PepO reprograms M2 macrophages towards M1 and to be tumoricidal. (A) Raw264.7 was co-cultured with PY8119 cells in the presence of PepO or not. The transcription level of M1 and M2 macrophage markers were measured using RT-qPCR. (B)The pro-inflammatory cytokine TNF-α and IL-6, and anti-inflammatory cytokine IL-10 in TAMs(Raw264.7) or TAMs treated by PepO were detected with ELISA kit. (C)The percentage of apoptotic PY8119 cells detected by FACS. (D) PY8119 cells were co-cultured with PBS, PepO control, and conditional medium (CM) of M0 BMDM or M0 BMDM treated by PepO, M2 BMDM or M2 BMDM treated by PepO, BMDM treated by PepO quivalents BSA and LPS. Then the apoptosis of 4T1 was assessed with annexin-V/PI kit and flow cytometry. (E) The expression of cleaved caspase-3(CC3) of PY8119 cells was evaluated via western blot. (F) RT-qPCR analysis was performed to examine the mRNA level of Cxcl9, Cxcl10 and Arg-1 in M2 phenotype BMDM and Raw264.7 treated by PepO or not. Two-way ANOVA with Tukey’s multiple comparisons test was used in (A)(n=3), and (F)(n=3). One-way ANOVA with Tukey’s multiple comparisons test was used in (B)(n=3) (C) and (D right)(n=3). Bar graphs represent mean ± SEM, *P<0.05,**P<0.01, ***P<0.001, ****P<0.0001. Figure S2. PepO inhibits TNBC growth in vivo. (A) Tumor growth profiles treated by high and low dose of PepO, and the tumor weights at the end of the experiment were recorded (n = 5). (B) The images of tumers of secondary tumorigenesis. (C) Western blot analysis of CSC marker expression in tumor tissue. (D) PY8119 cell migration was assessed with the woundhealing assay via co-culturing with conditioned medium. (E) Serum levels of ALT, AST, CREA, ALB, ALKP, CK of tumor-free C57BL/6 treated with PBS control or PepO(n=3). (F) Body weight of tumor-free C57BL/6 treated by PBS or PepO. (G) Representative H&E-stained sections of heart, liver, spleen, lung, kidney, or bone marrow. Serum and tissues were collected 4 days after the [file 13578_2023_1153_MOESM1_ESM.pdf]

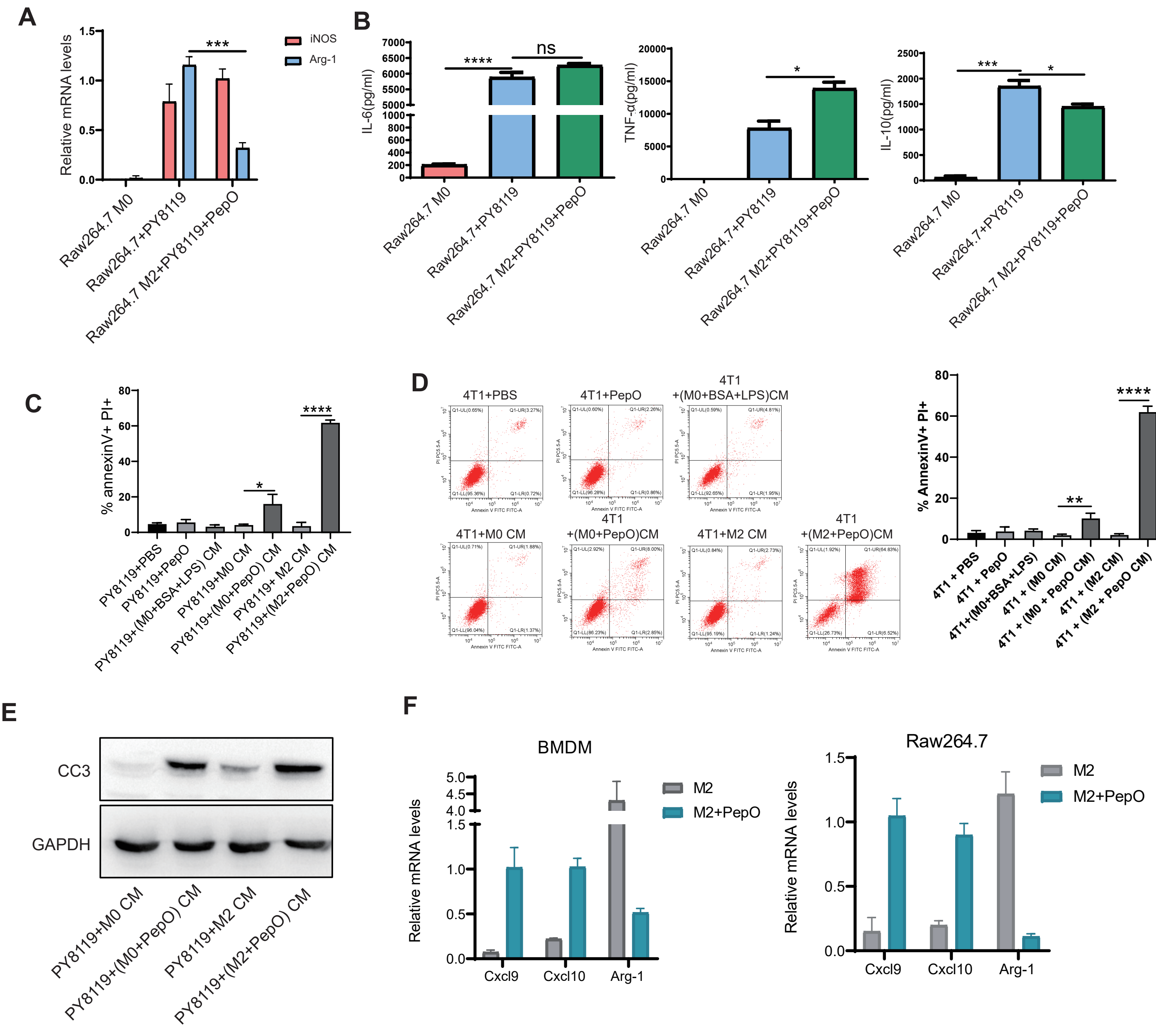

Figure S1. PepO reprograms M2 macrophages towards M1 and to be tumoricidal

(A) Raw264.7 was cocultured with PY8119 cells in the presence of PepO or not. The transcription level of M1 and M2 macrophage markers were measured using RT-qPCR. (B)The pro-inflammatory cytokine TNF-α and IL-6, and anti-inflammatory cytokine IL-10 in TAMs(Raw264.7) or TAMs treated by PepO were detected with ELISA kit. (C)The percentage of apoptotic PY8119 cells detected by FACS. (D) PY8119 cells were cocultured with PBS, PepO control, and conditional medium (CM) of M0 BMDM or M0 BMDM treated by PepO, M2 BMDM or M2 BMDM treated by PepO, BMDM treated by PepO quivalents BSA and LPS. Then the apoptosis of 4T1 was assessed with annexin-V/PI kit and flow cytometry. (E) The expression of cleaved caspase-3(CC3) of PY8119 cells was evaluated via western blot. (F) RT-qPCR analysis was performed to examine the mRNA level of Cxcl9, Cxcl10 and Arg-1 in M2 phenotype BMDM and Raw264.7 treated by PepO or not.

Two-way ANOVA with Tukey's multiple comparisons test was used in (A)(n=3), and (F)(n=3). One-way ANOVA with Tukey's multiple comparisons test was used in (B)(n=3) (C) and (D right)(n=3). Bar graphs represent mean ± SEM, *P<0.05,**P<0.01, ***P<0.001, ****P<0.0001.

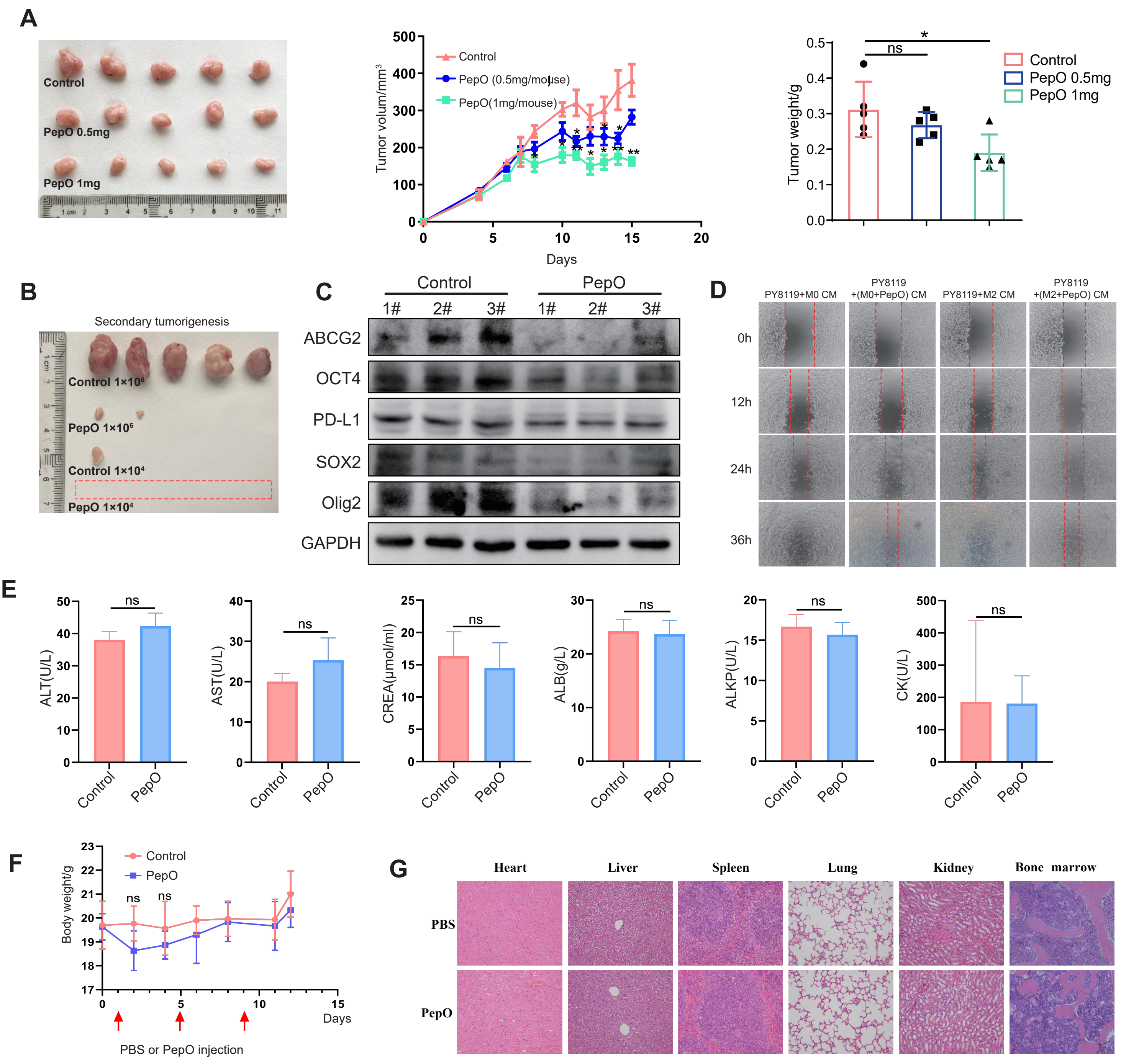

Figure S2 PepO inhibits TNBC growth in vivo.

(A) Tumor growth profiles treated by high and low dose of PepO, and the tumor weights at the end of the experiment were recorded (n = 5). (B) The images of tumers of secondary tumorigenesis. (C) Western blot analysis of CSC marker expression in tumor tissue. (D) PY8119 cell migration was assessed with the woundhealing assay via coculturing with conditioned medium. (E) Serum levels of ALT, AST, CREA, ALB, ALKP, CK of tumor-free C57BL/6 treated with PBS control or PepO(n=3). (F) Body weight of tumor-free C57BL/6 treated by PBS or PepO. (G) Representative H&E-stained sections of heart, liver, spleen, lung, kidney, or bone marrow. Serum and tissues were collected 4 days after the third treatment.

Two-way repeated-measures ANOVA with Sidak's multiple comparisons test was used in (A, Tumor growth profiles) and (F), and one-way ANOVA with Tukey's multiple comparisons test was used in (A, tumor weights). Two-tailed Student t test was used in (E). Bar graphs represent mean ± SEM.

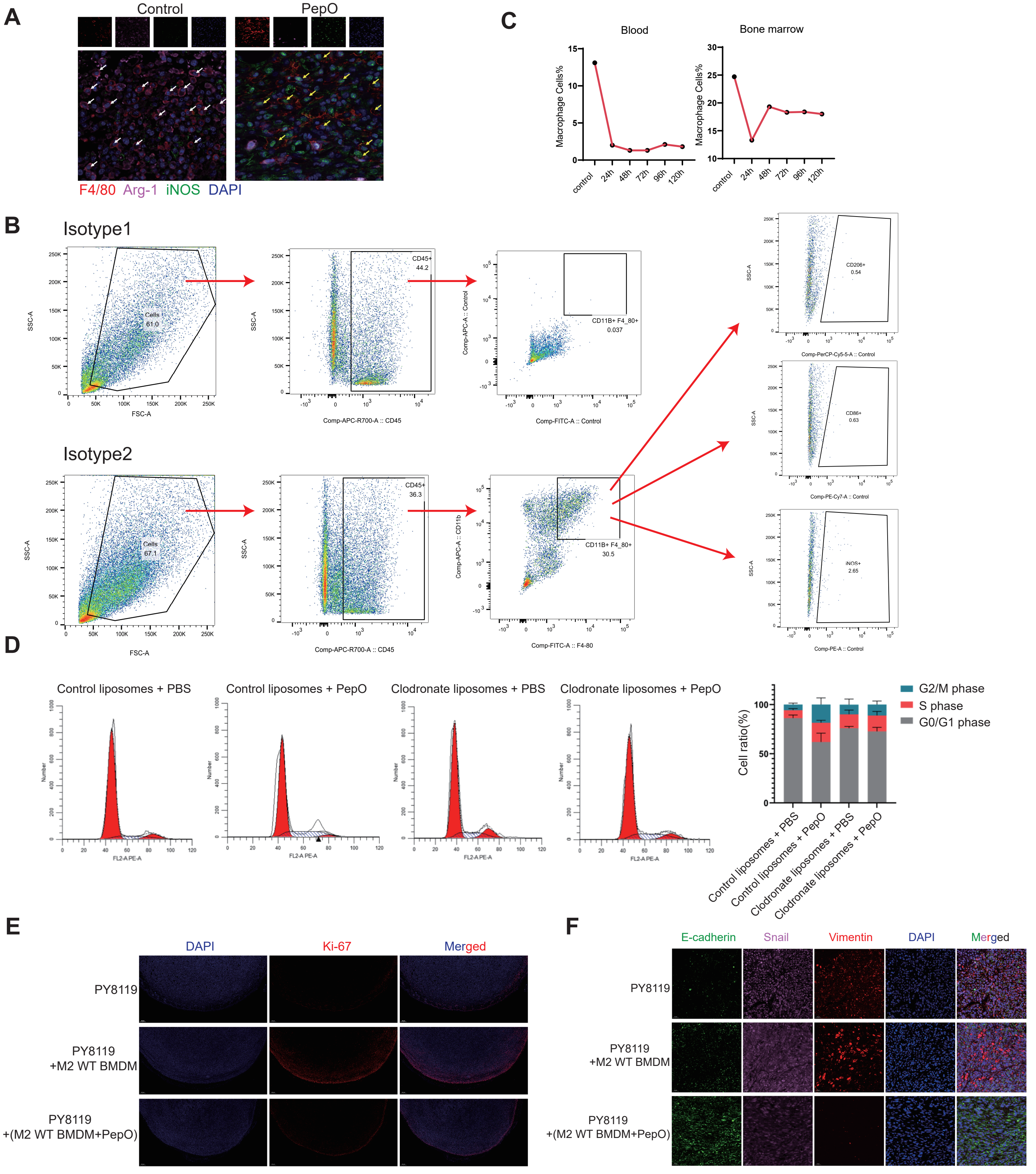

Figure S3 PepO reprograms TAMs and TAMs acquire anti-tumor capability in vivo

(A)Immunofluorescent triple staining for macrophage markers F4/80 (red), Arg-1 (pink), iNOS (green) in TNBC tissue established by PY8119. (B) Gating strategy for flow cytometry of macrophage subsets (Isotype1 was stained with mouse APC-R700-labelled CD45 antibody and APC mouse IgG1 κ Isotype Ctrl and FITC mouse IgG1 κ Isotype Ctrl; Isotype2 was stained with mouse APC-R700-labelled CD45 antibody, mouse FITC-labelled F4/80 antibody, mouse APC-labelled CD11b antibody and perCP/cy5.5 Rat IgG2a κ Isotype, PE/Cyanine7 Rat IgG1, λ Isotype Ctrl and PE Rat IgG2b κ Isotype Ctrl). (C)The efficiency of macrophages depletion in blood, bone marrow. (D) Flow cytometry was used to analize the phase of the tumor cell cycle from tumor tissue grouped as shown. (E-F) Cell proliferation (Ki-67), and EMT markers of TNBC established in nude mice was evaluated (scale bar of Ki-67=200μm, scale bar of EMT markers=20μm).

A

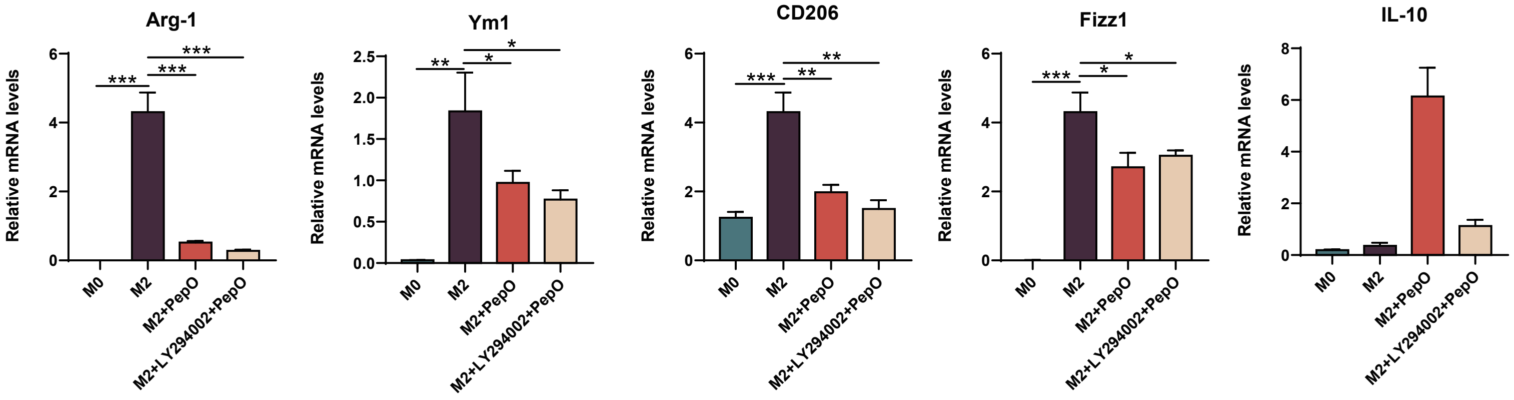

Figure S4 PepO reprogramed macrophages to be tumoricidal through activating PI3K-AKT- mTOR signaling pathway.

(A) The transcription level of M2 markers were measured by RT-qPCR.

One-way ANOVA with Tukey's multiple comparisons test was used in (A). Bar graphs represent mean ± SEM, *P<0.05, **P<0.01, ***P<0.001.

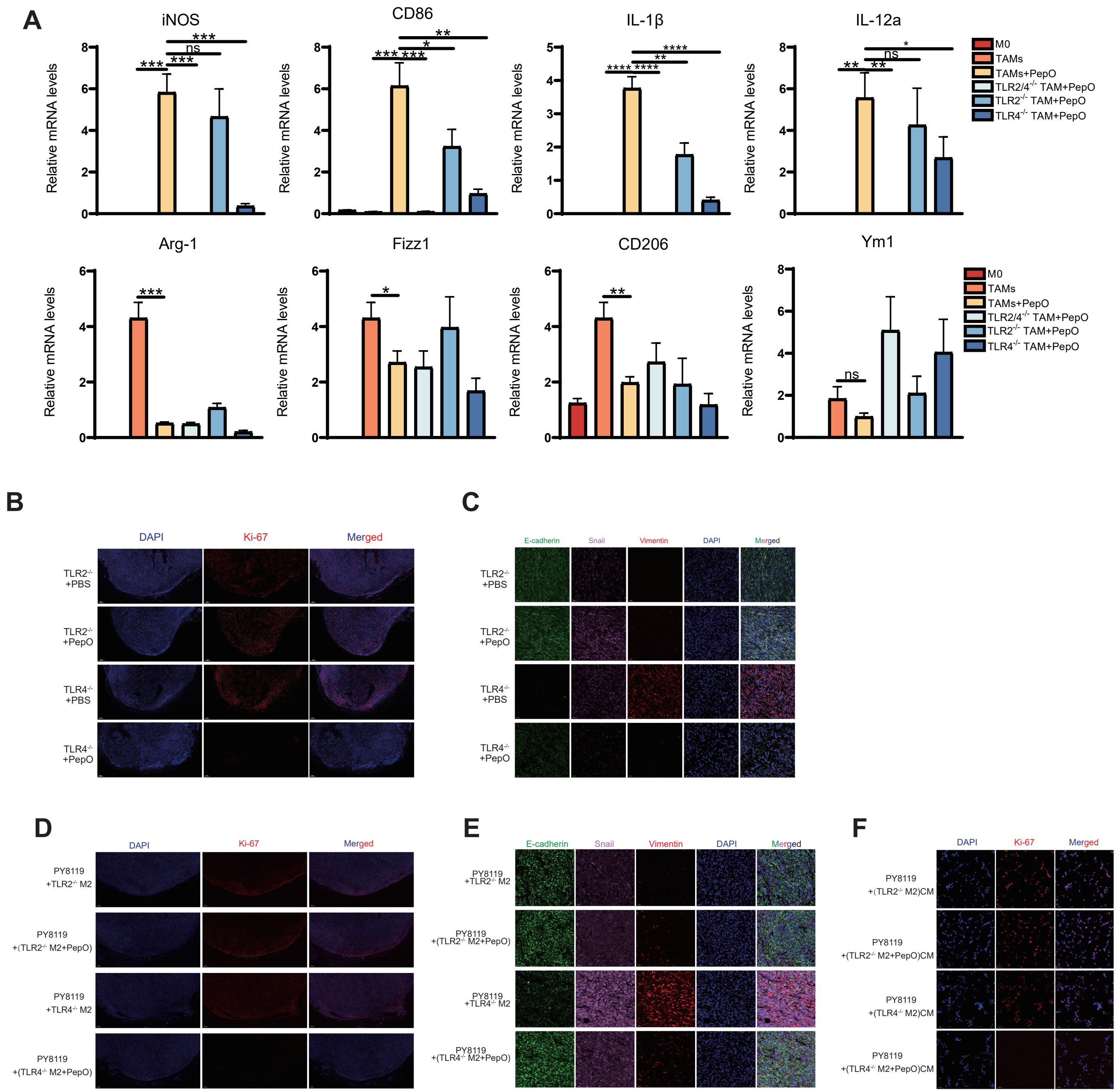

Figure S5 PepO reprogramed M2-like macrophage via being recognized by TLR2 and TLR4.
(A)The transcription level of macrophage markers were determined by RT-qPCR in indicated groups shown in A. (B) Cell proliferation (Ki-67) was evaluated using Immunofluorescence detection(scale bar=20μm). (C) Immunofluorescent triple staining for E-cadherin (green), Snail (pink), Vimentin (red) in TNBC tissue grouped as shown (scale bar=20μm). (D-E) The cancer cell proliferation (D, scale bar=50μm) and EMT process(E， scale bar=50μm) of nude mice grouped as labeled was assessed and the representative images were shown. (F) PY8119 cell were co-cultured with PepO- or PBS-primed gene deficient M2 BMDM, and the proliferation of PY8119 cell were evaluated by Ki-67.
One-way ANOVA with Tukey's multiple comparisons test was used in (A).

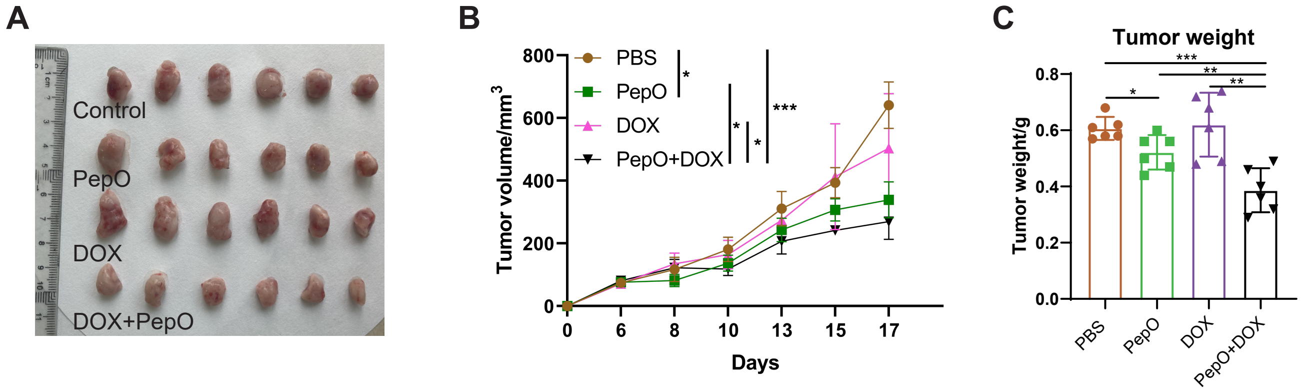

Figure S6 PepO combined with doxorubicin further inhibited tumor growth.
(A-C) PY8119 bearing C57BL/6 mice were treated with PBS, PepO, doxorubicin (2mg/kg), and the combination. The images of tumer (A), the tumor growth profiles (B) and tumor weight (C) were collected as shown. Two-way repeated-measures ANOVA with Sidak's multiple comparisons test was used in (B). One-way ANOVA with Tukey's multiple comparisons test was used in (C). Bar graphs represent mean ± SEM, *P<0.05, **P<0.01, ***P<0.001.
